# The Glycobiome of the Rumen Bacterium *Butyrivibrio proteoclasticus* B316^T^ Highlights Adaptation to a Polysaccharide-Rich Environment

**DOI:** 10.1371/journal.pone.0011942

**Published:** 2010-08-03

**Authors:** William J. Kelly, Sinead C. Leahy, Eric Altermann, Carl J. Yeoman, Jonathan C. Dunne, Zhanhao Kong, Diana M. Pacheco, Dong Li, Samantha J. Noel, Christina D. Moon, Adrian L. Cookson, Graeme T. Attwood

**Affiliations:** 1 Rumen Microbial Genomics, AgResearch, Grasslands Research Centre, Palmerston North, New Zealand; 2 Institute of Molecular Biosciences, Massey University, Palmerston North, New Zealand; 3 Centre for Biodiscovery and School of Biological Sciences, Victoria University of Wellington, Wellington, New Zealand; Universidad Miguel Hernandez, Spain

## Abstract

Determining the role of rumen microbes and their enzymes in plant polysaccharide breakdown is fundamental to understanding digestion and maximising productivity in ruminant animals. *Butyrivibrio proteoclasticus* B316^T^ is a Gram-positive, butyrate-forming rumen bacterium with a key role in plant polysaccharide degradation. The 4.4Mb genome consists of 4 replicons; a chromosome, a chromid and two megaplasmids. The chromid is the smallest reported for all bacteria, and the first identified from the phylum Firmicutes. B316 devotes a large proportion of its genome to the breakdown and reassembly of complex polysaccharides and has a highly developed glycobiome when compared to other sequenced bacteria. The secretion of a range of polysaccharide-degrading enzymes which initiate the breakdown of pectin, starch and xylan, a subtilisin family protease active against plant proteins, and diverse intracellular enzymes to break down oligosaccharides constitute the degradative capability of this organism. A prominent feature of the genome is the presence of multiple gene clusters predicted to be involved in polysaccharide biosynthesis. Metabolic reconstruction reveals the absence of an identifiable gene for enolase, a conserved enzyme of the glycolytic pathway. To our knowledge this is the first report of an organism lacking an enolase. Our analysis of the B316 genome shows how one organism can contribute to the multi-organism complex that rapidly breaks down plant material in the rumen. It can be concluded that B316, and similar organisms with broad polysaccharide-degrading capability, are well suited to being early colonizers and degraders of plant polysaccharides in the rumen environment.

## Introduction

The growth and productivity of ruminant animals depend on a complex microbial community located in their fore-stomach (rumen) which is able to breakdown plant polysaccharides and ferment the released sugars. The diet of forage-fed ruminants consists largely of structural polysaccharides that make up the plant cell wall, and storage polysaccharides such as starch and fructan. Plant cell walls have a basic structure of cellulose microfibrils surrounded by a complex matrix of hemicellulose, pectin and protein, with the composition varying between different plant species, cell types and stages of maturity [1]. Cellulose is the main component (35–50%) and consists of parallel β-1-4 glucan chains, whereas hemicellulose and pectin are branched polysaccharides whose structural complexity enables them to cross-link the cellulose microfibrils and form covalent bonds with other cell wall components such as lignin. The breakdown of plant cell walls, therefore, requires the coordinated action of diverse enzymes able to cleave a wide range of different chemical bonds.

The rumen microbiota consists mainly of obligately anaerobic microorganisms including bacteria, fungi, protozoa and methanogenic archaea that act together to achieve rapid breakdown of these complex plant polysaccharides. Cultivation studies have focused on the importance of the cellulolytic bacteria *Fibrobacter succinogenes*, *Ruminococcus albus* and *R. flavefaciens*, and the usual model of ruminal fibre breakdown places these in the central role as primary degraders, supported by xylanolytic *Butyrivibrio* and *Prevotella* species [2]. However, recent data obtained using cultivation independent techniques challenge this model. Direct amplification and sequencing of 16S ribosomal RNA genes [3] indicate that the majority of sequences are derived from organisms that are phylogenetically distinct from currently cultivated species implying that the diversity of organisms involved in plant polysaccharide breakdown in the rumen is only beginning to be revealed.

Central to the breakdown of plant cell walls are those bacteria that can adhere to plant material [4]. Studies focused on the adherent microbiota have revealed bacterial populations clearly distinct from those found in the planktonic fraction, and dominated by Firmicutes [5]–[7]. The adherent fraction displayed a high level of bacterial diversity but the commonly cultivated cellulolytic species were not often detected. Metagenomic sequence analysis of three fibre-adherent rumen microbiomes also showed a dominance of Firmicutes [8]. A gene-centric analysis was used to assess the prevalence of enzymes involved in polysaccharide degradation [8], and showed that although large numbers of glycoside hydrolases could be detected, very few of these belonged to families known to hydrolyse intact cellulose, hemicellulose or pectin. This led the authors to propose a different model for fibre degradation in which initial colonization is by organisms that remove the easily available side chains of complex plant polysaccharides [8]. These bacteria are subsequently replaced by organisms that can degrade the main chains of cellulose and hemicellulose.

*Butyrivibrio proteoclasticus* B316^T^ (formerly *Clostridium proteoclasticum*) [9], [10] is a Gram positive, polysaccharide-degrading, butyrate-producing, anaerobic bacterium isolated from the bovine rumen. Taxonomically it belongs in the clostridial rRNA subcluster XIVa and is a member of the family *Lachnospiraceae* [11]. Studies from New Zealand and Finland using quantitative molecular techniques have shown *B. proteoclasticus* to be present at high numbers in the rumen contents from animals consuming pasture or grass silage based diets [12], [13], and it has also been commonly detected in rumen 16S rDNA libraries [3]. Because of its wide distribution among ruminant animals consuming a variety of diets, its ability to degrade plant proteins and polysaccharides, and to biohydrogenate fatty acids [14], *B. proteoclasticus* was chosen for genome sequencing. Here we report the complete genome sequence of the type strain of *B. proteoclasticus* (B316^T^ = ATCC 51982^T^ = DSM 14932^T^), a bacterium that devotes a large proportion of its genome to the breakdown and reassembly of complex polysaccharides (glycobiome). Although genomic information is available for some rumen fibrolytic bacteria [15], [16] this is the first reported complete genome sequence.

## Results

### General genome features

**Figure 1:**
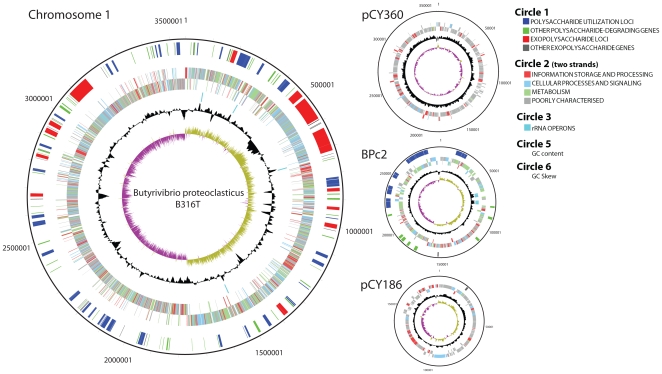
Genome atlas highlighting the glycobiome of *Butyrivibrio proteoclasticus* B316. The figure shows the four replicons that make up the B316 genome and the location of the genes predicted to encode proteins involved in polysaccharide degradation and exopolysaccharide biosynthesis. The four rRNA operons on the main chromosome and two on BPc2 are also shown. The colour coding of the genomic features in circle 2 represent different Clusters of Orthologous Groups (COG) categories.

The major features of the 4.4 Mb *B. proteoclasticus* B316^T^ genome are presented in Table 1 and Fig. 1. The B316 genome encodes 3813 coding sequences (CDSs) spread over four replicons; the main chromosome, a chromid [17], and two megaplasmids. BPc2 is the smallest chromid reported and and the first identified from the phylum Firmicutes [17]. The COG (Clusters of Orthologous Groups of proteins) distribution of BPc2 is very similar to the main chromosome (Fig. S1), but differs markedly from the two megaplasmids which mainly encode proteins of unknown function that show little homology to database sequences. The nucleotide sequence of the *Butyrivibrio proteoclasticus* B316^T^ genome has been deposited in Genbank under Accession Numbers CP001810 (main chromosome), CP001811 (BPc2), CP001812 (pCY360) and CP001813 (pCY186).

**Table 1:** Features of each of the four replicons that make up the *Butyrivibrio proteoclasticus* B316 genome.

	BPc1^1^	BPc2^1^	pCY360	pCY186	Total
Size (bp)	3 554 804	302 358	361 399	186 325	4 404 886
G+C%	40.21	40.04	38.95	38.10	39.33
CDSs	2939	251	425	198	3813
rRNAs	4	2	0	0	6
tRNAs	47	2	0	1	50
GHs^2^	98	15	0	0	113
GTs^2^	120	0	0	1	121

1 BPc1 and BPc2 refer to the main chromosome and the chromid respectively.

2 GHs, glycoside hydrolases; GTs, glycosyl transferases.

A novel feature of the B316 genome is that the order of the genes within the rRNA operons is 16S-5S-23S whereas bacterial rRNA genes are predominantly arranged in the order 16S-23S-5S. Examination of complete (n = 3) and draft (n = 21) genome sequences available for other members of the *Lachnospiraceae* showed that the 16S-5S-23S arrangement is common to this bacterial family.

### The B316 glycobiome

**Figure 2:**
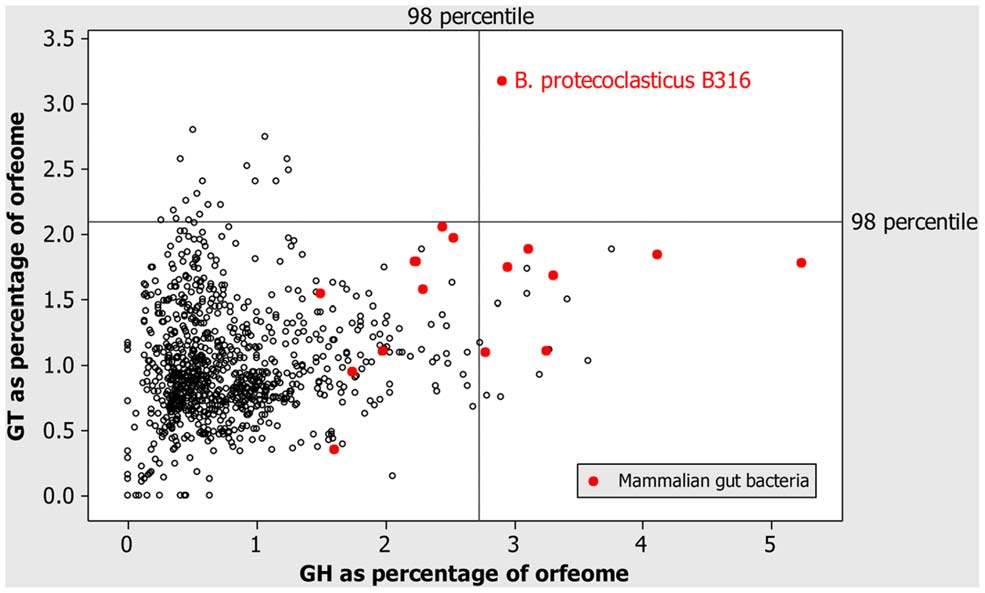
Comparison of bacterial glycobiomes. Comparative analysis of the glycoside hydrolase and glycosyl transferase complement of B316 against bacterial genomes included in the CAZy database (n = 960).

The main feature of the B316 genome sequence, is its extensive repertoire of genes predicted to encode enzymes involved in polysaccharide degradation and reassembly (Fig. 1 and Table S1). The glycoside hydrolase and glycosyl transferase content of the B316 glycobiome was compared with results for bacteria recorded in the CAZy (Carbohydrate-active enzymes) database (http://www.cazy.org/) (n = 960) as of February 2010 (Fig. 2) and B316 has a highly developed glycobiome compared to the majority of other sequenced bacteria. Bacteria from the human gut show similar extensive repertoires of GH and GT genes [18], and it is apparent that this is a feature of bacteria from gastrointestinal environments. The total number of different CAZy families represented in the B316 genome (Table S2) surpasses that found in the cellulolytic rumen bacteria *F. succinogenes* and *R. flavefaciens* [16] and in the fibre-adherent rumen metagenome [8].

### Polysaccharide breakdown

B316 is unable to degrade crystalline cellulose but uses a range of other plant polysaccharides including inulin, pectin, starch and xylan [9], [10]. Degradation of these insoluble plant polysaccharides requires bacteria to adhere to the substrate and for enzymes to be secreted from the cell. Bacteria have evolved various strategies to manage this process [7] with some rumen cellulose-degrading bacteria producing a multi-enzyme cellulosome complex in which enzymes are linked to a non-catalytic scaffold structure via dockerin domains [19]. The cellulosome producing *R. flavefaciens* FD-1 secretes 75% of its glycoside hydrolases and most of these are predicted to be cellulosome-associated [16]. In contrast, B316 secretes only a third of its polysaccharide degrading enzymes (Table S1), none of which contain dockerin domains. B316 also lacks the scaffoldin/cohesin components involved in cellulosome assembly. A group of nine large cell-associated proteins (Xyn10B, Amy13A, Lic16A, Xsa43J, Agn53A, Pme8B, Est12B, Pel1A and Bpr_I0264) represent the core of the catalytic capability of B316 and can initiate the breakdown of pectin, starch and xylan. These proteins all contain multiple cell wall binding repeat domains (Pfam accession number PF01473) at their C-termini which are believed to anchor them to the peptidoglycan cell wall. These cell wall binding domains are common in peptidoglycan hydrolases from Gram positive bacteria [20], but this is the first report of their association with enzymes involved in plant polysaccharide breakdown. The catalytic and carbohydrate binding regions of Amy13A are similar (>50% amino acid identity) to the cell-associated α-amylase (Amy13B) from the human isolate *B. fibrisolvens* 16/4, but there is no homology in the C-terminal region [21]. The 16/4 amylase contains a hydrophobic region and a short basic C-terminus preceded by a partially conserved LPXTG motif used in sortase-mediated cell wall attachment. Therefore, catalytically similar enzymes from phylogenetically related gut bacteria may use different mechanisms for attaching enzymes to their cell surfaces. The retention of these hydrolytic enzymes at the B316 cell surface is likely to favor rapid uptake of released oligosaccharides, most of which is mediated by a range of ABC transporters.

Many of the secreted carbohydrate degrading enzymes also contain non-catalytic carbohydrate-binding modules (CBMs) which mediate the association of the enzyme with its substrate. CBMs have been subdivided into three types based on structural and functional similarities. Type A bind to highly crystalline cellulose, type B bind to glycan chains while type C bind optimally to mono-, di- or tri-saccharides [22]. B316 encodes CBMs belonging to all three types typified by CBM2a, CBM6 and CBM13 domains respectively (Table S1). Mxy10-43A has all three CBM types together with GH10 and GH43 domains making it a particularly versatile enzyme. CBM2 has previously been subdivided into two subfamilies, 2a and 2b, based on a Arg/Gly polymorphism that confers specificity for xylan or cellulose [23], and alignment of the CBMs from the B316 enzymes clearly grouped them with CBM2a (Fig. S2). CBM2a is able to bind to crystalline cellulose and to primary and secondary plant cell walls [24], can move along the cellulose surface [25], and can disrupt cellulose structure to release non-covalently attached fragments [26]. B316 has nine copies of CBM2a found in Cel5B, Cel5C, Cel9C, Xyn10C, Mxy10-43A with tandem copies in two carbohydrate-binding proteins that lack catalytic domains (Bpr_I0736, Bpr_I1599). The likely function of these carbohydrate-binding proteins is to improve access to plant structural polysaccharides.

Other secreted enzymes that do not degrade polysaccharides may also contribute to plant polysaccharide breakdown. Animal feeding experiments and *in vitro* studies [27], [28] have shown increased fibre digestibility following supplementation with a subtilisin-like protease feed additive, which was postulated to act by hydrolyzing cell wall structural proteins or protein-polysaccharide crosslinks and allowing better access for fibrolytic microbes [28]. B316 produces a cell-bound serine protease activity [9] attributed to a secreted subtilisin family serine protease (Bpr_I2629). It is possible that this protease has a specialized role, acting synergistically with a range of carbohydrate-degrading enzymes to improve access to the polysaccharide substrate.

Two-thirds of the B316 enzymes involved in polysaccharide degradation are not secreted from the cell, and contain few carbohydrate-binding modules. Of the most prevalent GH families (Table S1), all the members of GH2 and GH31 are intracellular along with most GH3, GH13 and GH43 enzymes. Of the enzymes predicted to be involved in pectin and xylan breakdown, those belonging to the GH8, GH28, GH39, GH51, GH67, GH88, GH105, GH115, CE2 and CE10 families are all intracellular implying that a variety of complex oligosaccharides resulting from extracellular hydrolysis are metabolized within the cell. The most prevalent GH families reported in the rumen metagenome [8], and in several other sequence-based metagenomic studies targeting different environments [29], include GH2, GH3, GH13, GH31, GH43 and GH51. The prevalence of intracellular enzymes in these groups suggests that function-based metagenomic studies may be better suited for the detection of secreted core polysaccharide degrading enzymes.

More than half of the genes encoding intracellular polysaccharide degrading proteins are clustered in polysaccharide utilization loci (PUL) which also include transporters, transcriptional regulators, environmental sensors such as two component system histidine kinase/response regulators and genes involved in further metabolism (Fig. 1 and Table S3). The clustering of genes involved in oligosaccharide breakdown appears to be a common strategy in both Gram negative [30] and Gram positive [16], [31] polysaccharide degrading bacteria presumably allowing coordinated control of enzyme production, substrate transport and intracellular metabolism.

Thirty two-component systems and many other proteins with sensory transduction domains were identified within the genome, several of which are located in the PULs. In addition, multiple copies of genes encoding sugar-responsive transcriptional regulators are present, 17 belonging to the AraC family, 15 belonging to the LacI family and one belonging to the DeoR family. Six response regulators involved in two-component systems with helix-turn helix AraC domains (Pfam accession number PF00165) and three response regulators with LytTR domains (Pfam accession number PF04397) were also present. These LytTR domain proteins appear to have a common function since they are each found close to a secreted GH3 family enzyme with a C-terminal TMH domain (PUL7, 21 and 33). In its rumen environment B316 is exposed to a range of carbohydrates, and therefore it is likely that these sensory transduction proteins and transcriptional regulators are used to detect the types of carbohydrate linkages present, and control expression of the appropriate enzymes.

Biohydrogenation is strongly associated with the adherent bacterial fraction [32], and it has been hypothesised that the process is a detoxification mechanism which facilitates fibre digestion. High levels of the polyunsaturated fatty acids (PUFAs) linolenic acid (C_18:3_) and linoleic acid (C_18:2_) [32] are present in fresh forage, and these are toxic to some rumen bacteria, particularly the cellulolytic ruminococci [33]. *Butyrivibrio* species are regarded as the main rumen bacteria that carry out biohydrogenation, and the only bacterium known to carry out the transformation of the toxic PUFAs through to the less toxic stearic acid (C18:0) is *B. proteoclasticus* [14].

### Metabolism of sugars derived from polysaccharide breakdown

**Figure 3:**
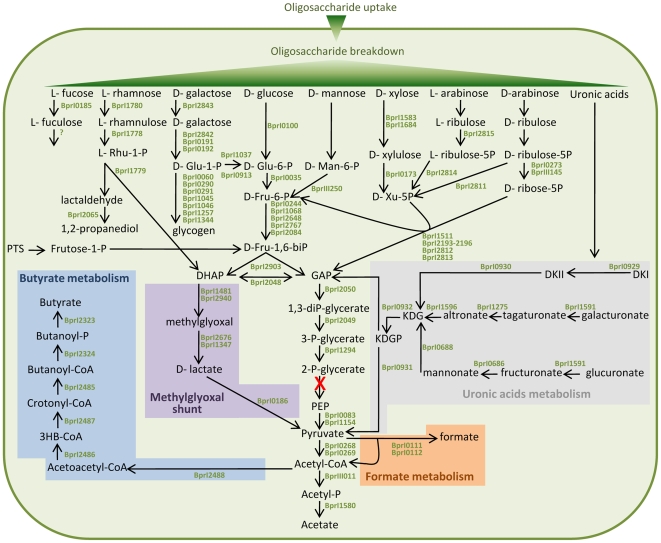
Overview of carbohydrate metabolism in *B. proteoclasticus* B316. The enolase catalysed reaction is shown in red as neither the gene nor the enzyme activity was present in B316. Abbreviations: DHAP, dihydroxyacetone phosphate; DKI, 5-keto-4-deoxyuronate; DKII, 2,5-diketo-3-deoxygluconate; GAP, glyceraldehyde-3-phosphate; 3HB-CoA, 3-hydroxybutanoyl-CoA; KDG, 2-keto-3-deoxygluconate; KDGP, 2-keto-3-deoxygluconate phosphate; PEP, phosphoenolpyruvate.

**Figure 4:**
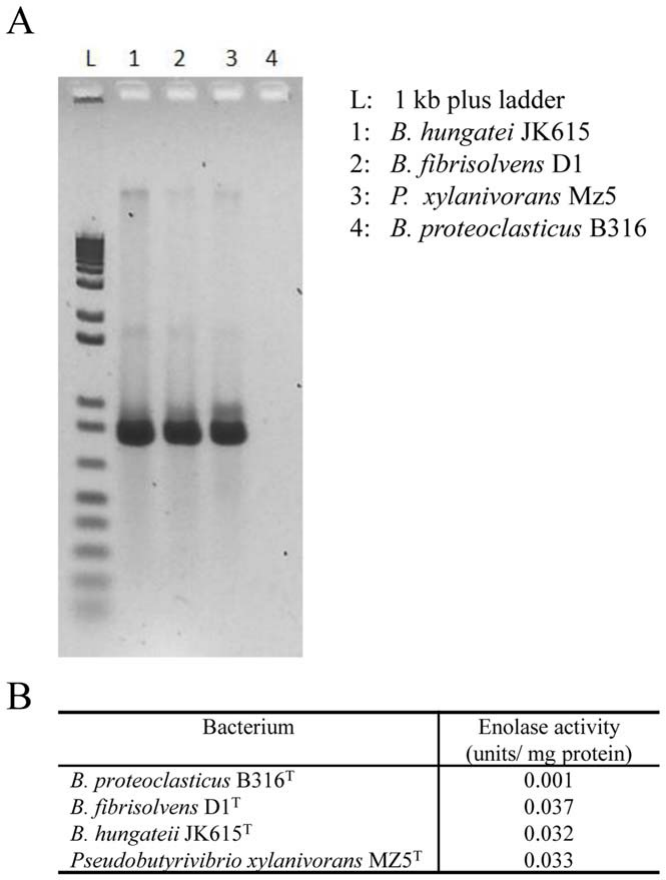
Enolase from *Butyrivibrio* and *Pseudobutyrivibrio*. A, PCR amplification of the enolase gene from *Butyrivibrio* and *Pseudobutyrivibrio* cultures using primers designed from the *B. crossotus* genome sequence. B, Enolase enzyme activity of *Butyrivibrio* and *Pseudobutyrivibrio* cultures.

Breakdown of plant polysaccharides should make several different sugars available to the cell and the predicted catabolic pathways are shown in Fig. 3, together with the pathways to the major fermentation end-products, butyrate and formate [9]. Surprisingly, B316 lacks an identifiable enolase (EC4.2.1.11), the enzyme that converts 2-phosphoglycerate to phosphoenolpyruvate in the penultimate step of the Embden-Meyerhof (EM) glycolytic pathway. Intact enolase genes can be found in the draft genome sequences of *Butyrivibrio crossotus* and other *Lachnospiraceae* but not in *B. fibrisolvens* 16/4 where the gene is truncated (Genbank accession number CBK73998). Degenerate PCR primers designed from the *B. crossotus* sequence were able to amplify a product from *B. fibrisolvens* D1^T^, *B. hungatei* JK615^T^ and *Pseudobutyrivibrio xylanivorans* Mz5^T^ (Fig. 4), but not from B316 (The nucleotide sequences of enolase gene fragments from *B. fibrisolvens* D1^T^, *B. hungateii* JK615^T^ and *Pseudobutyrivibrio xylanivorans* MZ5^T^ were deposited in Genbank under Accession Numbers GU937437-GU937439). Coupled enzyme assays showed that enolase activity was present in these *Butyrivibrio* and *Pseudobutyrivibrio* strains but only a very low level was detected in B316 (Fig. 4).

A possible alternative to the EM pathway is the methylglyoxal shunt (Fig. 3) by which dihydroxyacetone phosphate (DHAP) is transformed to pyruvate via methylglyoxal and D-lactate [34]. B316 has two methylglyoxal synthases (Bpr_I1481 and Bpr_I2940), together with enzymes that show homology to glyoxalase I (glyoxalase family protein, Bpr_I2676), glyoxalase II (metallo-beta-lactamase family protein, Bpr_I1347) and D-lactate dehydrogenase (D-isomer specific 2-hydroxyacid dehydrogenase, Bpr_I0186). Use of the methylglyoxal shunt has been proposed as a strategy by which rumen bacteria can dispose of excess carbohydrate and decrease ATP production [35], but the operation of this pathway as an alternative to glycolysis in *B. proteoclasticus* awaits further confirmation. The possibility of the rumen bacteria *B. fibrisolvens* and *R. albus* having an uncommon pathway from triosephosphate to pyruvate has been raised previously [36], as enolase and pyruvate kinase activity could not be detected, or was extremely low, in cell-free extracts of these species.

Breakdown of pectin, and of arabinoxylan substituted with alpha-glucuronic acid which is typically found in grasses, makes galacturonates and glucuronates available for further metabolism. The EM pathway is not useful for degradation of galacturonates and glucuronates as there is no enzymatic conversion through to glucose-6-phosphate. Their metabolism (Fig. 3) occurs through a common intermediate, 2-keto-3-deoxygluconate (KDG), which is converted to 2-keto-3-deoxygluconate phosphate (KDGP) by 2-dehydro-3-deoxygluconokinase (Bpr_I0932). KDGP is then converted to pyruvate and glyceraldehyde-3-phosphate (GAP) by 2-keto-3-deoxygluconate 6-phosphate aldolase (Bpr_I00931). A metabolic footprint of B316 grown on xylan, pectin and glucose [37] showed that levels of xylose, rhamnose and β-arabinoside increased in the media, but that galacturonic acid decreased suggesting that any galacturonic acid released is quickly consumed. Consequently, galacturonates and glucuronates available as pectin and xylan breakdown products are important growth substrates for B316, and a major source of pyruvate for central metabolism. This argument is strengthened by the observation that all except one (Bpr_I1275) of the genes involved in glucuronate metabolism are clustered in PULs (PUL7, 9 and 13) and likely to be coordinated with other genes involved in polysaccharide breakdown.

### Polysaccharide reassembly

One problem faced by B316 is what to do with excess carbohydrate following the metabolism of oligosaccharides transported into the cell [35]. One solution is to recycle the component sugars and reassemble them into different polysaccharides which can then be used for other purposes.

When grown in liquid media B316 produces copious quantities of a ropy exopolysaccharide (EPS, Fig. S3A), and EPS is also seen covering cells grown on plant material (Fig. S3B). Most *Butyrivibrio* strains produce EPS, and differences in their sugar composition have been used for taxonomic discrimination [38]. Although the composition of the EPS from B316 has not been determined, the neutral sugars present in EPS from other *Butyrivibrio* strains include rhamnose, fucose, mannose, galactose and glucose [38], all of which would be available from the recycling of degraded plant polysacccharides.

A total of 363 genes are predicted to be involved in polysaccharide biosynthesis, arranged in 13 exopolysaccharide loci (EPSL) located on the main chromosome (Fig. 1 and Table S4). Seven EPSL contain 132 genes with notably lower G+C% than the rest of the genome, and three of these are adjacent to transposases (EPSL1, 7 and 8) suggesting they may have been acquired via horizontal gene transfer events. EPSL3 and 4 are separated by a cluster of flagellar biosynthesis genes (Bpr_I0474-I0489) and similar gene arrangements, known as flagellar glycosylation islands, are found in several other bacteria that produce glycosylated flagella [39]. Flagella produced by *Pseudobutyrivibrio ruminis* OR77 (formerly *B. fibrisolvens*) have been reported to be glycosylated [40].

Electron microscopy of B316 shows the presence of cytoplasmic inclusions (Fig. S3D), similar to those seen in other *Butyrivibrio* strains containing glycogen-like material [41]. Chemical analysis showed that carbohydrate made up 11% of the cell dry mass of B316, and the genome encodes a full complement of genes for the synthesis and degradation of glycogen. The presence of both glycogen biosynthetic and degradative capability suggests that glycogen acts as a storage polysaccharide in B316, but it may also have a role in the regulation of excess carbohydrate.

### Adherence

The ability to adhere to plant material is an essential attribute for ruminal plant polysaccharide degrading bacteria, and B316 has several potential mechanisms for adhering to plant cell walls. These include the CBM-containing proteins and exopolysaccharides together with flagella, pili and cell surface proteins. In addition, B316 has been observed to adopt a filamentous cell morphology (Fig. S3E and F) as has been previously reported for *B. fibrisolvens* [42].

B316 has a single sub-terminal flagellum, and possesses 46 genes involved in flagellar assembly and function, but is non-motile, in contrast to other closely related bacteria. Flagellar gene organization is very similar to that found in other low GC Gram positive bacteria with most of the genes arranged in two clusters. The larger gene cluster, which encodes the early expressed genes that make up the flagella basal body and hook, the type III secretion system and motility functions, is disrupted by a ferulic acid esterase gene (*est*1B, Bpr_I1368) inserted next to *flg*E and immediately upstream of the genes for two motility proteins MotA and MotB. Despite its lack of motility B316 encodes an extensive repertoire of genes for sensing and responding to changes in the environment, including 14 methyl-accepting chemotaxis proteins and a variety of other chemotaxis proteins and sensory transducers. It is possible that B316 uses its flagellum for adherence rather than motility. Flagella have been observed to mediate the symbiotic association between syntrophic bacteria and methanogens [43], and B316 interacts with the ruminal methanogen *Methanobrevibacter ruminantium* M1 enabling H_2_ or formate to be transferred [44]. To examine which genes are involved in this interaction, B316 was grown in co-culture with M1 and gene expression analysed by microarrays [44]. Twelve of the genes found in the larger flagellar gene cluster were significantly upregulated (>2-fold, FDR <0.05), together with one of the flagellin genes (*fli*C2) located between EPSL3 and EPSL4 (The microarray data have been deposited in NCBI's Gene Expression Omnibus (GEO) in accordance with MIAME standards and are accessible through GEO Series accession GSE18716).

B316 produces pili (Fig. S3C) and prepilin peptidases flank genes predicted to be involved in a type IV secretion system for pilus production (Bpr_I0064-I0072). The pilus structural proteins have not been identified, although there is a cluster of genes including those for a signal peptidase, three LPxTG motif-containing surface proteins and two sortases (Bpr_I0893-I0898) that shows similar gene organization to pilus islands found in other Gram positive bacteria [45].

In addition to these complex surface structures B316 also encodes a small number of cell surface proteins. These include four proteins (Bpr_I2082, I2426, I2508 and II321) with *Listeria/Bacteroides* repeats (TIGRfam accession number TIGR02543), and one large protein (Bpr_I0752, 3385 aa) with a C-terminal TMH predicted to anchor the protein to the cell membrane. In the microarray experiment described above, the gene showing the highest level of up-regulation (19.1 fold) encodes a secreted protein of unknown function containing a von Willebrand factor type A domain (Bpr_I0925).

## Discussion

The B316 genome has a number of novel features including atypical rRNA gene order and a multi-replicon genome architecture which includes the smallest chromid to be reported for bacteria, together with two megaplasmids whose function is largely unknown. The megaplasmids are hypothesised to encode properties important for survival in the rumen environment, but they make no detectable contribution to the glycobiome. The B316 genome encodes a large repertoire of glycoside hydrolases and glycosyl transferases (Fig. 2) and when genes associated in loci associated with polysaccharide utilization (Table S3) and exopolysaccharide production (Table S4) are included, more than 20% of the genome (>760 CDSs) is devoted to the glycobiome (Fig. 1). The presence of a gene does not automatically mean that it is expressed and we are currently determining which genes and proteins are upregulated during growth on particular plant polysaccharides. Furthermore, the rumen contains a complex microbial community and there are undoubtedly many interactions that occur between the various species present.

The plant cell wall-microbe-enzyme interface is at the centre of ruminal polysaccharide breakdown and this study shows how one organism can contribute to the multi-organism complex that rapidly breaks down plant material. The incomplete EM pathway, the predicted use of the methylglyoxal shunt as an alternative to glycolysis, the production of intra- and extracellular polysaccharides and the prediction that glucuronate and galacturonate derived from xylan and pectin are important growth substrates can all be seen as specialised adaptations to the polysaccharide-rich rumen environment. Identification of the complex B316 glycobiome is a step towards understanding ruminant digestion and will not only contribute to mapping the complex conversion of plant biomass into milk and meat for human consumption, but also give an improved choice of enzymes for improving fibre digestion in ruminant animals. Information on the strategies used by rumen bacteria to breakdown plant cell walls is also likely to be relevant to initiatives seeking to develop cost-effective enzyme-based processes for converting biomass to fuels and chemicals.

## Materials and Methods

### Bacterial strains and growth media

*Butyrivibrio proteoclasticus* B316^T^, *B. crossotus* DSM 2876, *B. fibrisolvens* D1^T^ (DSM 3071), *B. hungateii* JK615^T^ (DSM 14810), *Pseudobutyrivibrio xylanivorans* MZ5^T^ (DSM 14809) were grown anaerobically under a CO_2_ atmosphere in DSM medium 704 (http://www.dsmz.de/media/med704.htm) at 39°C.

### Genome sequencing and assembly

A whole genome shotgun strategy (Agencourt Biosciences, USA) and a pyrosequencing approach (454 Life Sciences, USA). A hybrid assembly [46] was performed utilising the Staden package [47], Phred [48], Phrap (http://www.phrap.org) and Repeatmasker (http://repeatmasker.org). Standard and long range PCR was used to close gaps, improve sequence quality and resolve any remaining base-conflicts. The genome assembly was confirmed by pulsed field gel electrophoresis (PFGE).

### Genome analysis and annotation

A GAMOLA [49]/Artemis [50] software suite was used to manage genome annotation. Coding sequences (CDSs) were identified and assigned functions as described previously [44]. Metabolic pathway reconstructions were performed using Pathway Voyager [51] and the KEGG (Kyoto Encyclopedia of Genes and Genomes) database [52] combined with an extensive review of the literature. Circular diagrams were created using circular_diagram.pl (Rutherford, K, Sanger Centre software). Genome sequence and orfeome (total number of CDSs) information for comparative studies was obtained from the NCBI (http://www.ncbi.nlm.nih.gov), MetaHIT (http://www.sanger.ac.uk/pathogens/metahit) and FibRumBa (http://www.jcvi.org/rumenomics). Comparative analysis of the B316 glycobiome involved the total number of glycoside hydrolase and glycosyl transferase enzymes for each genome recorded in the CAZy (Carbohydrate-active enzymes) database (http://www.cazy.org/) being tabulated and calculated as a % of the orfeome. A scatterplot was created using MINITAB® Release 15 (Minitab Inc., USA).

### Microscopy

Light microscopy of B316 cells was carried out using a Leica model DM2500. Electron microscopy of negatively stained cells and of thin sections was carried out with a Philips model 201C electron microscope. Whole-cell preparations were negatively stained with 1% phosphotungstic acid and mounted on Formvar-coated copper grids. Thin sections were prepared from bacterial cell pellets as previously described [9]. For scanning electron microscopy (SEM), samples were gold coated before viewing with a Jeol JSM 7000F Field Emission Gun scanning electron microscope.

### Enolase primers and activity assay

Screening of *Butyrivibrio* species for enolase genes was carried out by PCR amplification using the following degenerate primers: forward 5′-aatggacctaYgcagatgc-3′ and reverse 5′-atctggttRagcttWataag-3′.

Enolase activity of *Butyrivibrio* cell-free extracts was assayed spectrophotometrically at 340 nm by coupling the reaction with pyruvate kinase and lactate dehydrogenase to give oxidation of NADH [53] and using reagents from Sigma-Aldrich (St. Louis, MO, USA).

### Glycogen storage

Intracellular glycogen was obtained by heating B316 cell pellets in 30% (w/v) potassium hydroxide for 3 h at 100°C, cooling to room temperature and precipitating polysaccharide material by the addition of two volumes of ethanol. The carbohydrate content of the precipitated material was estimated by the phenol sulfuric acid assay [54].

## Supporting Information

Figure S1 COG distribution of each of the four replicons that make up the B. proteoclasticus B316 genome.
(0.99 MB TIF)

Figure S2 Sequence alignment of CBM 2 families from B. proteoclasticus B316. The sequence numbering refers to the B316 CDSs with the exception of cenA; Cellulomonas fimi endoglucanase A (accession number P07984)-CBM2a family, and xylD1; C.fimi xylanase D (accession number P54865)-CBM2b family, representative of the two classes of CBM domains. Conserved tryptophans, which are believed to be the main sites of polysaccharide interaction, are shown in blue. The glycine and arginine residues that confer specificity for cellulose or xylan are shown in green and red respectively.
(1.12 MB TIF)

Figure S3 Electron microscopy and light microscopy of B316. A, Transmission EM of B316 cells grown in liquid medium. B, Scanning EM of B316 cells growing on a clover leaf surface. C, Transmission EM of a thin section of a B316 cell showing the presence of pili. D, Transmission EM of a thin section of a B316 cell showing the presence of glycogen inclusions. E, Light microscopy of a B316 culture showing normal growth morphology. F, Light microscopy of a B316 culture showing the filamentous growth morphology.
(6.20 MB TIF)

Table S1 B. proteoclasticus B316 ORFs encoding enzymes and binding proteins involved in polysaccharide degradation.
(0.15 MB DOC)

Table S2 Diversity of plant polysaccharide degradation capabilities in rumen fibrolytic bacteria compared with the rumen metagenome.
(0.03 MB DOC)

Table S3 Polysaccharide utilization loci (PUL) from the genome of B. proteoclasticus B316.
(0.08 MB DOC)

Table S4 Exopolysaccharide biosynthesis loci (EPSL) from the genome of B. proteoclasticus B316.
(0.07 MB DOC)

## References

[1] Cosgrove DJ (2005). Growth of the plant cell wall.. Nat Rev Mol Cell Bio.

[2] Flint HJ, Bayer EA, Rincon MT, Lamed R, White BA (2008). Polysaccharide utilization by gut bacteria: potential for new insights from genomic analysis.. Nat Rev Microbiol.

[3] Edwards JE, McEwan NR, Travis AJ, Wallace RJ (2004). 16S rRNA library-based analysis of ruminal bacterial diversity.. Antonie van Leeuwenhoek.

[4] Edwards JE, Huws SA, Kim EJ, Kingston-Smith AH (2007). Characterization of the dynamics of initial bacterial colonization of nonconserved forage in the bovine rumen.. FEMS Microbiol Ecol.

[5] Koike S, Yoshitani S, Kobayashi Y, Tanaka K (2003). Phylogenetic analysis of fiber-associated rumen bacterial community and PCR detection of uncultured bacteria.. FEMS Microbiol Lett.

[6] Larue R, Yu Z, Parisi VA, Egan AR, Morrison M (2005). Novel microbial diversity adherent to plant biomass in the herbivore gastrointestinal tract, as revealed by ribosomal intergenic spacer analysis and *rrs* gene sequencing.. Environ Microbiol.

[7] Morrison M, Pope PB, Denman SE, McSweeney CS (2009). Plant biomass degradation by gut microbiomes: more of the same or something new?. Curr Opin Biotech.

[8] Brulc JM (2009). Gene-centric metagenomics of the fiber-adherent bovine rumen microbiome reveals forage specific glycoside hydrolases.. Proc Natl Acad Sci USA.

[9] Attwood GT, Reilly K, Patel BKC (1996). *Clostridium proteoclasticum* sp. nov., a novel proteolytic bacterium from the bovine rumen.. Int J Syst Bacteriol.

[10] Moon CD (2008). Reclassification of *Clostridium proteoclasticum* as *Butyrivibrio proteoclasticus* comb. nov., a butyrate-producing ruminal bacterium.. Int J Syst Evol Microbiol.

[11] Ludwig W, Schleifer K-H, Whitman WB (2008). Revised road map to the phylum *Firmicutes*.. http://www.bergeys.org/outlines/Bergeys_Vol_3_Outline.pdf.

[12] Reilly K, Attwood GT (1998). Detection of *Clostridium proteoclasticum* and closely related strains in the rumen by competitive PCR.. Appl Environ Microbiol.

[13] Paillard D (2007). Quantification of ruminal *Clostridium proteoclasticum* by real-time PCR using a molecular beacon approach.. J Appl Microbiol.

[14] Wallace RJ (2006). *Clostridium proteoclasticum*: a ruminal bacterium that forms stearic acid from linoleic acid.. FEMS Microbiol Lett.

[15] Jun HS, Qi M, Ha JK, Forsberg CW (2007). *Fibrobacter succinogenes*, a dominant fibrolytic ruminal bacterium: transition to the post genomic era.. Asian-Aust J Anim Sci.

[16] Berg Miller ME (2009). Diversity and strain specificity of plant cell wall degrading enzymes revealed by the draft genome of *Ruminococcus flavefaciens* FD-1.. PLoS ONE.

[17] Harrison PW, Lower RPJ, Kim NKD, Young JPW (2010). Introducing the bacterial ‘chromid’:not a chromosome, not a plasmid.. Trends Microbiol.

[18] Lozupone CA (2008). The convergence of carbohydrate active gene repertoires in human gut microbes.. Proc Natl Acad Sci USA.

[19] Bayer EA, Lamed R, White BA, Flint HJ (2008). From cellulosomes to cellulosomics.. Chem Rec.

[20] Layec S, Decaris B, Leblond-Bourget N (2008). Diversity of Firmicutes peptidoglycan hydrolases and specificities of those involved in daughter cell separation.. Res Microbiol.

[21] Ramsay AG, Scott KP, Martin JC, Rincon MT, Flint HJ (2006). Cell-associated α-amylases of butyrate-producing Firmicute bacteria from the human colon.. Microbiol.

[22] Boraston AB, Bolam DN, Gilbert HJ, Davies GJ (2004). Carbohydrate-binding modules: fine-tuning polysaccharide recognition.. Biochem J.

[23] Simpson PJ, Xie H, Bolam DN, Gilbert HJ, Williamson MP (2000). The structural basis for the ligand specificity of family 2 carbohydrate-binding modules.. J Biol Chem.

[24] Blake AW (2006). Understanding the biological rationale for the diversity of cellulose-directed carbohydrate-binding modules in prokaryotic enzymes.. J Biol Chem.

[25] Liu Y-S (2009). Does the cellulose-binding module move on the cellulose surface?. Cellulose.

[26] Din N (1994). C1-Cx revisited: intramolecular synergism in a cellulase.. Proc Natl Acad Sci USA.

[27] Eun J-S, Beauchemin KA (2005). Effects of a proteolytic feed enzyme on intake, digestion, ruminal fermentation, and milk production.. J Dairy Sci.

[28] Colombatto D, Beauchemin KA (2009). A protease additive increases fermentation of alfalfa diets by mixed ruminal microorganisms in vitro.. J Anim Sci.

[29] Li L-L, McCorkle SR, Monchy S, Taghavi S, van der Lelie D (2009). Bioprospecting metagenomes: glycosyl hydrolases for converting biomass.. Biotechnol Biofuels.

[30] Martens EC, Koropatkin NM, Smith TJ, Gordon JI (2009). Complex glycan catabolism by the human gut microbiota: the Bacteroidetes sus-like paradigm.. J Biol Chem.

[31] Van de Werken HJG (2008). Hydrogenomics of the extremely thermophilic bacterium *Caldicellulosiruptor saccharolyticus*.. Appl Environ Microbiol.

[32] Kim EJ, Sanderson R, Dhanoa MS, Dewhurst RJ (2005). Fatty acid profiles associated with microbial colonization of freshly ingested grass and rumen biohydrogenation.. J Dairy Sci.

[33] Maia MRG, Chaudhary LC, Figueres L, Wallace RJ (2006). Metabolism of polyunsaturated fatty acids and their toxicity to the microflora of the rumen.. Antonie van Leeuwenhoek.

[34] Cooper RA (1984). Metabolism of methylglyoxal in microorganisms.. Ann Rev Microbiol.

[35] Russell JB (1998). Strategies that ruminal bacteria use to handle excess carbohydrate.. J Anim Sci.

[36] Kistner A, Kotzé JP (1973). Enzymes of intermediary metabolism of *Butyrivibrio fibrisolvens* and *Ruminococcus albus* grown under glucose limitation.. Can J Microbiol.

[37] Villas-Bôas SG, Noel S, Lane GA, Attwood G, Cookson A (2006). Extracellular metabolomics: a metabolic footpprinting approach to assess fiber degradation in complex media.. Anal Biochem.

[38] Stack RJ (1988). Neutral sugar composition of extracellular polysaccharides produced by strains of *Butyrivibrio fibrisolvens*.. Appl Environ Microbiol.

[39] Logan SM (2006). Flagellar glycosylation-a new component of the motility repertoire?. Microbiol.

[40] Kalmokoff ML (2000). Biochemical and genetic characterization of the flagellar filaments from the rumen anaerobe *Butyrivibrio fibrisolvens* OR77.. Anaerobe.

[41] Hespell RB, Kato K, Costerton JW (1993). Characterization of the cell wall of *Butyrivibrio* species.. Can J Microbiol.

[42] Sewell GW (1988). Isolation and characterization of xylan-degrading strains of *Butyrivibrio fibrisolvens* from a napier grass-fed anaerobic digester.. Appl Environ Microbiol.

[43] Shimoyama T, Kato S, Ishii S, Watanabe K (2009). Flagellum mediates symbiosis.. Science.

[44] Leahy SC (2010). The genome sequence of the rumen methanogen *Methanobrevibacter ruminantium* reveals new possibilities for controlling ruminant methane emissions.. PLoS ONE.

[45] Mandlik A, Swierczynski A, Das A, Ton-That H (2008). Pili in Gram-positive bacteria: assembly, involvement in colonization and biofilm development.. Trends Microbiol.

[46] Goldberg SM (2006). A Sanger/pyrosequencing hybrid approach for the generation of high-quality draft assemblies of marine microbial genomes.. Proc Natl Acad Sci USA.

[47] Staden R, Beal KF, Bonfield JK (2000). The Staden package, 1998.. Methods Mol Biol.

[48] Ewing B, Hillier L, Wendl MC, Green P (1998). Base-calling of automated sequencer traces using phred. I. Accuracy assessment.. Genome Res.

[49] Altermann E, Klaenhammer TR (2003). GAMOLA: a new local solution for sequence annotation and analyzing draft and finished prokaryotic genomes.. Omics.

[50] Rutherford K (2000). Artemis: sequence visualization and annotation.. Bioinformatics.

[51] Altermann E, Klaenhammer TR (2005). PathwayVoyager: pathway mapping using the Kyoto Encyclopedia of Genes and Genomes (KEGG) database.. BMC Genomics.

[52] Kanehisa M, Goto S (2000). KEGG: Kyoto Encyclopedia of Genes and Genomes.. Nucleic Acids Res.

[53] Bergmeyer HU (1974). Methods in Enzymatic Analysis, 2nd ed.,.

[54] Ashwell G (1957). Colorimetric analysis of sugars.. Methods Enzymol.

