# Supplementary figures and images for: The Glycobiome of the Rumen Bacterium Butyrivibrio proteoclasticus B316T Highlights Adaptation to a Polysaccharide-Rich Environment
Source: PLoS One. 2010 Aug 3;5(8):e11942. doi: 10.1371/journal.pone.0011942 (PMC2914790; doi:10.1371/journal.pone.0011942)

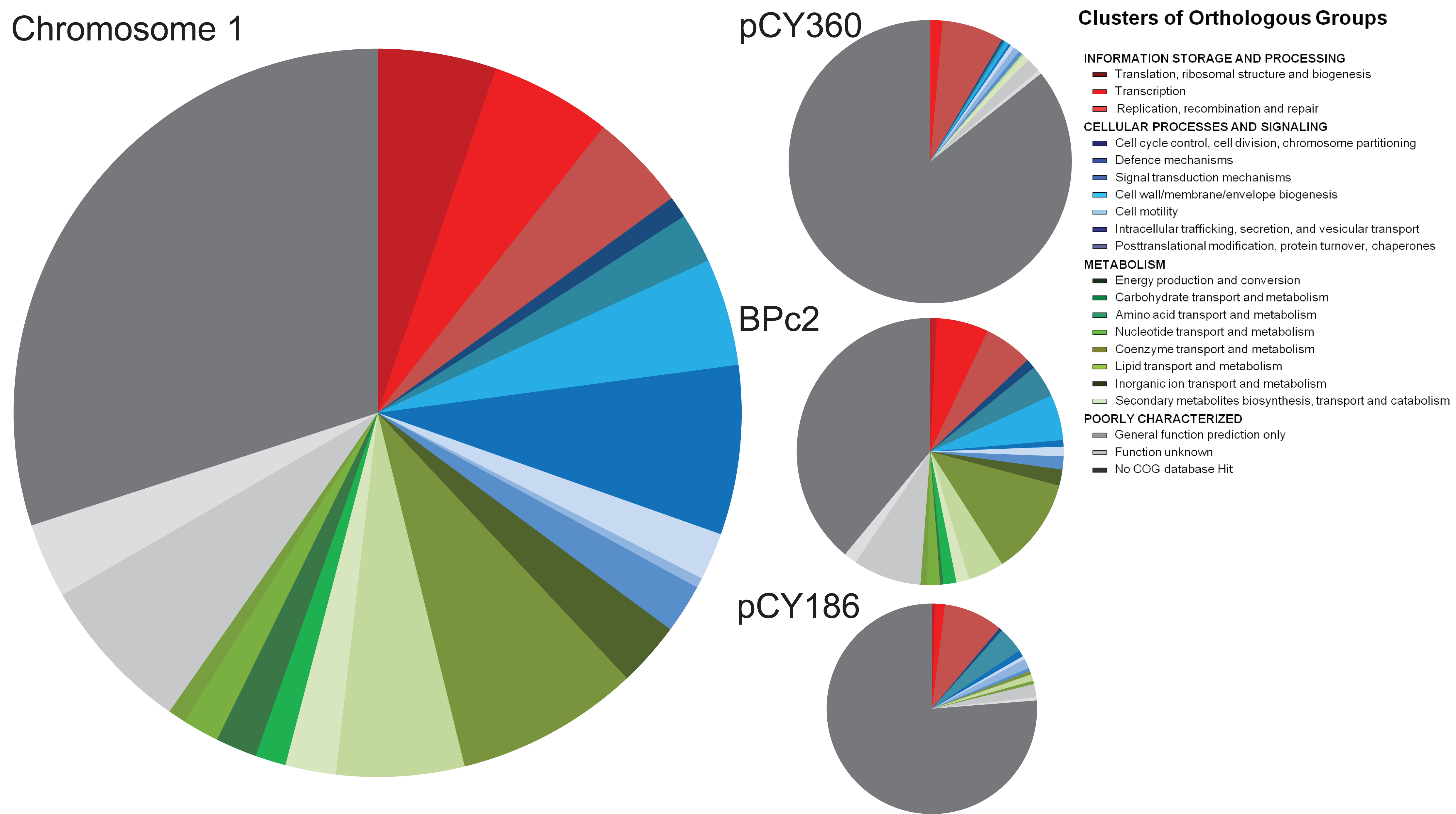

Supplement: Figure S1 — COG distribution of each of the four replicons that make up the B. proteoclasticus B316 genome. (0.99 MB TIF) [file pone.0011942.s001.tif]

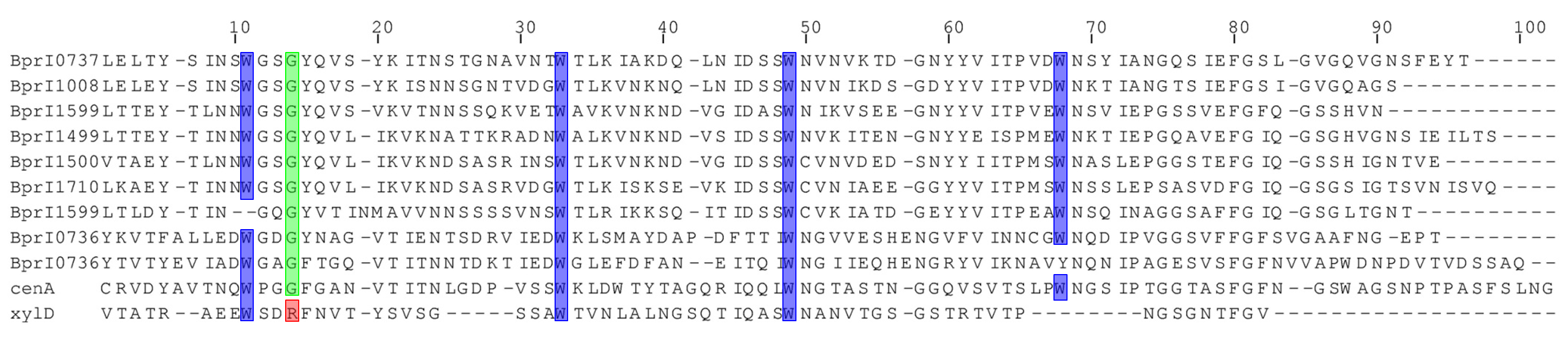

Supplement: Figure S2 — Sequence alignment of CBM 2 families from B. proteoclasticus B316. The sequence numbering refers to the B316 CDSs with the exception of cenA; Cellulomonas fimi endoglucanase A (accession number P07984)-CBM2a family, and xylD1; C.fimi xylanase D (accession number P54865)-CBM2b family, representative of the two classes of CBM domains. Conserved tryptophans, which are believed to be the main sites of polysaccharide interaction, are shown in blue. The glycine and arginine residues that confer specificity for cellulose or xylan are shown in green and red respectively. (1.12 MB TIF) [file pone.0011942.s002.tif]

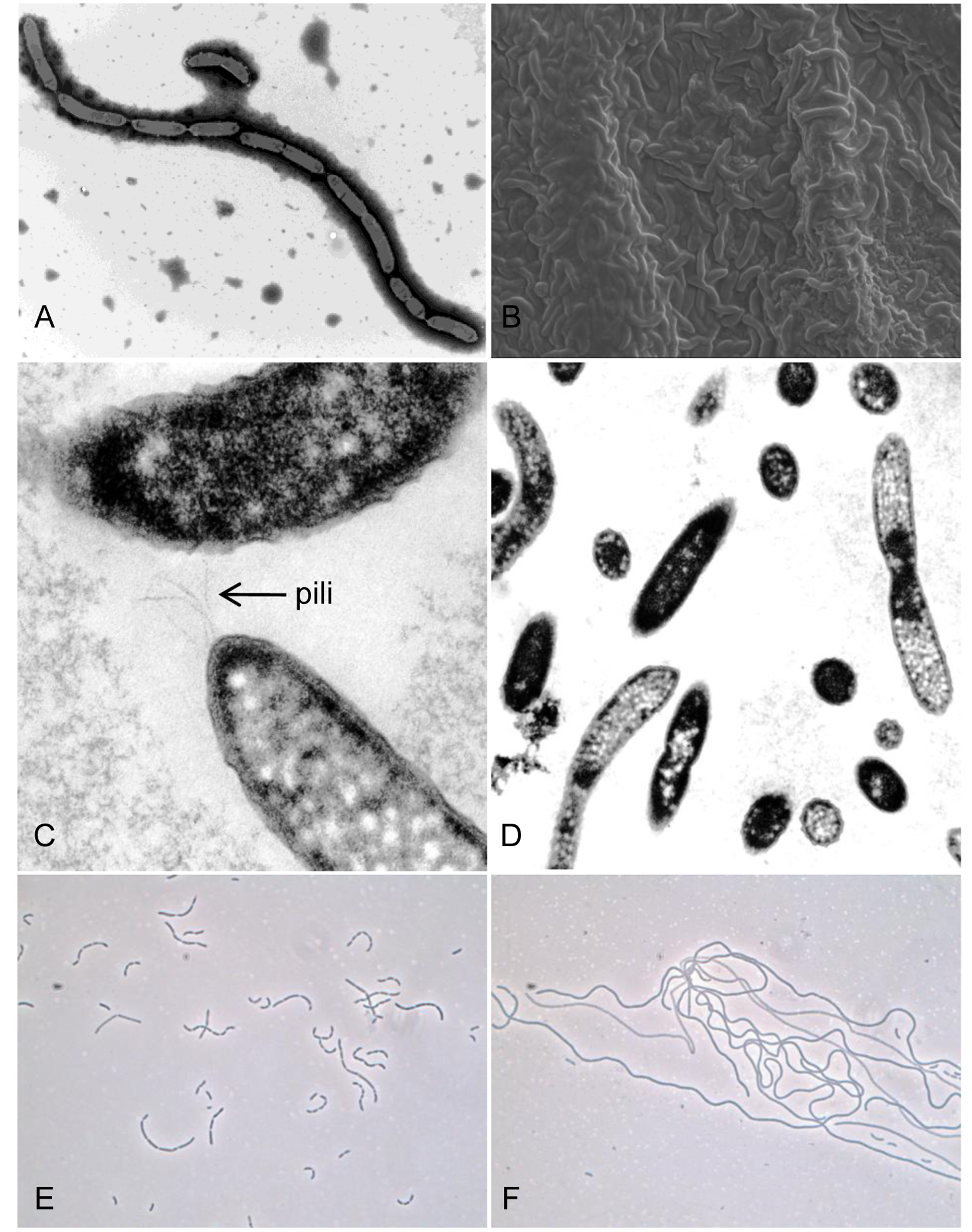

Supplement: Figure S3 — Electron microscopy and light microscopy of B316. A, Transmission EM of B316 cells grown in liquid medium. B, Scanning EM of B316 cells growing on a clover leaf surface. C, Transmission EM of a thin section of a B316 cell showing the presence of pili. D, Transmission EM of a thin section of a B316 cell showing the presence of glycogen inclusions. E, Light microscopy of a B316 culture showing normal growth morphology. F, Light microscopy of a B316 culture showing the filamentous growth morphology. (6.20 MB TIF) [file pone.0011942.s003.tif]
