# Supplementary material for: The Glycobiome of the Rumen Bacterium Butyrivibrio proteoclasticus B316T Highlights Adaptation to a Polysaccharide-Rich Environment
Source: PLoS One. 2010 Aug 3;5(8):e11942. doi: 10.1371/journal.pone.0011942 (PMC2914790; doi:10.1371/journal.pone.0011942)
Supplement: Table S1 — B. proteoclasticus B316 ORFs encoding enzymes and binding proteins involved in polysaccharide degradation. (0.15 MB DOC) [file pone.0011942.s004.doc]

Table S1. *B. proteoclasticus* B316 CDSs encoding enzymes and binding proteins involved in polysaccharide degradation.

| 1. Secreted proteins | | | | |
| --- | --- | --- | --- | --- |
| Locus tag | Gene name | Size (aa) | Catalytic domains | Binding domains^1^ |
| Bpr_I0693 | beta-glucosidase Bgl3A | 1067 | GH3 | C-terminal TMH |
| Bpr_I1795 | beta-N-acetylhexosaminidase Bhx3B | 426 | GH3 |  |
| Bpr_I2096 | beta-glucosidase Bgl3D | 972 | GH3 | C-terminal TMH |
| Bpr_III208 | beta-N-acetylhexosaminidase Bhx3C | 666 | GH3 |  |
| Bpr_III218 | glycoside hydrolase family 3 Gh3A | 982 | GH3 | C-terminal TMH |
| Bpr_I0299 | endo-1,4-beta-glucanase/xylanase Cel5A | 456 | GH5 |  |
| Bpr_I0728 | endo-1,4-beta-glucanase Cel5D | 503 | GH5 |  |
| Bpr_I1499 | endo-1,4-beta-glucanase Cel5B | 616 | GH5 | CBM2a |
| Bpr_I1710 | endo-1,4-beta-glucanase Cel5C | 547 | GH5 | CBM2a |
| Bpr_I1500 | endo-1,4-beta-glucanase Cel9C | 825 | GH9 | CBM2a, CBM3 |
| Bpr_I2796 | endo-1,4-beta-glucanase Cel9A | 530 | GH9 |  |
| Bpr_I0026 | endo-1,4-beta-xylanase Xyn10B | 1233 | GH10 | DUF1083, CW (x3) |
| Bpr_I0304 | endo-1,4-beta-xylanase Xyn10A | 476 | GH10 |  |
| Bpr_I0737 | endo-1,4-beta-xylanase and xylosidase Mxy10-43A | 1394 | GH10, GH43 | CBM2a, CBM6 (x2), CBM13 |
| Bpr_I1008 | endo-1,4-beta-xylanase Xyn10C | 996 | GH10, CE1 | CBM2a, CBM13 |
| Bpr_I1740 | endo-1,4-beta-xylanase Xyn10E | 434 | GH10 |  |
| Bpr_I0777 | alpha-amylase Amy13B | 647 | GH13 |  |
| Bpr_I1087 | alpha-amylase Amy13A | 1262 | GH13 | CBM26 (x2), CW (x7) |
| Bpr_III161 | pullulanase Pul13A | 906 | GH13 | CBM41, CBM48, PUD, C-terminal TMH |
| Bpr_I2326 | endo-1,3(4)-beta-glucanase Lic16A | 1381 | GH16 | CW (x5) |
| Bpr_I0271 | chitinase Chi18A | 567 | GH18 |  |
| Bpr_I0190 | lysozyme Lyc25C | 567 | GH25 | CW (x9) |
| Bpr_I1326 | lysozyme Lyc25B | 486 | GH25 |  |
| Bpr_I1569 | lysozyme Lyc25A | 362 | GH25 |  |
| Bpr_I2937 | glycoside hydrolase family 30 Gh30A | 607 | GH30 |  |
| Bpr_I1953 | glycoside hydrolase family 32 Gh32B | 512 | GH32 |  |
| Bpr_I0302 | xylosidase/arabinofuranosidase Xsa43A | 536 | GH43 | CBM6 |
| Bpr_I0675 | xylosidase/arabinofuranosidase Xsa43F | 490 | GH43 |  |
| Bpr_I2935 | xylosidase/arabinofuranosidase Xsa43J | 2284 | GH30, GH43 | Big4 (x2), FIVAR, CW (x7) |
| Bpr_I2041 | arabinogalactan endo-1,4-beta-galactosidase Agn53A | 1109 | GH53 | Big4, CW (x7) |
| Bpr_III093 | arabinogalactan endo-1,4-beta-galactosidase Agn53B | 709 | GH53 | Big4 |
| Bpr_I0587 | endo-1,3-beta-glucanase Glu55A | 586 | GH55 |  |
| Bpr_I2429 | 4-alpha-glucanotransferase Mal77A | 507 | GH77 |  |
| Bpr_I2480 | alpha-L-rhamnosidase Rha78C | 1037 | GH78 |  |
| Bpr_I0187 | feruloyl esterase Est1C | 370 | CE1 |  |
| Bpr_I1844 | feruloyl esterase Est1A | 374 | CE1 |  |
| Bpr_I0951 | polysaccharide deacetylase Est4C | 334 | CE4 |  |
| Bpr_I2473 | pectin methylesterase Pme8B | 2732 | CE8 | CW (x9) |
| Bpr_I1204 | carbohydrate esterase family 12 Est12B | 2676 | CE12, PL11 | Big4 (x3), fn3 (x2), FG (x4), CW (x7) |
| Bpr_I2372 | pectate lyase Pel1A | 1062 | PL1 | CW (x9) |
| Bpr_III190 | pectate lyase Pel9A | 510 | PL9 | CBM13 |
| Bpr_I0264 | cell wall binding domain-containing protein^2^ | 1802 |  | CW (x7) |
| Bpr_I0736 | carbohydrate binding protein | 523 |  | CBM2a (x2) |
| Bpr_I1599 | carbohydrate binding protein | 708 |  | CBM2a (x2), CBM6 (x2) |
| 2. Intracellular proteins | | | | |
| Bpr_I1685 | beta-glucosidase Bgl1A | 434 | GH1 |  |
| Bpr_I0175 | glycoside hydrolase family 2 Gh2C | 1174 | GH2 |  |
| Bpr_I0199 | glycoside hydrolase family 2 Gh2B | 646 | GH2 |  |
| Bpr_I0279 | beta-galactosidase Bga2A | 1039 | GH2 |  |
| Bpr_I0680 | glycoside hydrolase family 2 Gh2D | 591 | GH2 |  |
| Bpr_I0691 | glycoside hydrolase family 2 Gh2A | 671 | GH2 |  |
| Bpr_I1687 | glycoside hydrolase family 2 Gh2E | 737 | GH2 |  |
| Bpr_I1699 | glycoside hydrolase family 2 Gh2F | 915 | GH2 |  |
| Bpr_III209 | beta-galactosidase Bga2B | 820 | GH2 |  |
| Bpr_III237 | beta-mannosidase Man2A | 824 | GH2 |  |
| Bpr_I0138 | beta-glucosidase Bgl3C | 825 | GH3 |  |
| Bpr_I0184 | beta-xylosidase Xyl3A | 709 | GH3 |  |
| Bpr_I0847 | beta-glucosidase Bgl3B | 938 | GH3 |  |
| Bpr_I0912 | beta-N-acetylhexosaminidase Bhx3A | 586 | GH3 |  |
| Bpr_I2095 | beta-glucosidase Bgl3E | 806 | GH3 |  |
| Bpr_I1513 | reducing end xylose-releasing exo-oligoxylanase Xyn8A | 380 | GH8 |  |
| Bpr_I1593 | cellodextrinase Cel9B | 547 | GH9 | CelD |
| Bpr_I1083 | endo-1,4-beta-xylanase and esterase Xyn10D | 692 | GH10, CE10 |  |
| Bpr_I0060 | 1,4-alpha-glucan branching enzyme GlgB1 | 639 | GH13 | CBM48 |
| Bpr_I0135 | alpha-alpha-phosphotrehalase TreC | 576 | GH13 |  |
| Bpr_I0700 | alpha-amylase Amy13F | 613 | GH13 |  |
| Bpr_I0729 | alpha-amylase Amy13G | 528 | GH13 |  |
| Bpr_I1232 | glycogen debranching enzyme GlgX1 | 725 | GH13 | CBM48 |
| Bpr_I1344 | 1,4-alpha-glucan branching enzyme GlgB2 | 845 | GH13 | CBM48 (x2) |
| Bpr_I1494 | glycogen debranching enzyme GlgX2 | 658 | GH13 |  |
| Bpr_I1840 | alpha-amylase Amy13C | 434 | GH13 |  |
| Bpr_I2261 | sucrose phosphorylase Suc13P | 552 | GH13 |  |
| Bpr_I2684 | alpha-amylase Amy13D | 558 | GH13 | CBM34 |
| Bpr_I2904 | alpha-amylase Amy13E | 696 | GH13 |  |
| Bpr_I1522 | endo-1,3(4)-beta-glucanase Lic16B | 252 | GH16 |  |
| Bpr_I0544 | lysozyme Lyc25E3 | 1225 | GH25 | Big2 (x5), CW |
| Bpr_I2126 | lysozyme Lyc25D^3^ | 533 | GH25 | SH3 (x2) |
| Bpr_I0090 | glycoside hydrolase family 27 Gh27A | 454 | GH27 |  |
| Bpr_I0205 | alpha-galactosidase Aga27A | 594 | GH27 |  |
| Bpr_I2505 | polygalacturonase Pgl28A | 524 | GH28 |  |
| Bpr_I2854 | polygalacturonase Pgl28B | 519 | GH28 |  |
| Bpr_III015 | alpha-L-fucosidase Fuc29A | 492 | GH29 |  |
| Bpr_I0701 | glycoside hydrolase family 31 Gh31B | 873 | GH31 |  |
| Bpr_I1018 | glycoside hydrolase family 31 Gh31A | 752 | GH31 |  |
| Bpr_I1974 | glycoside hydrolase family 31 Gh31C | 674 | GH31 |  |
| Bpr_I2860 | glycoside hydrolase family 31 Gh31D | 652 | GH31 |  |
| Bpr_III199 | glycoside hydrolase family 31 Gh31E | 807 | GH31 |  |
| Bpr_I1703 | sucrose-6-phosphate hydrolase Scr32A | 493 | GH32 |  |
| Bpr_I1704 | glycoside hydrolase family 32 Gh32A | 414 | GH32 |  |
| Bpr_I0938 | beta-galactosidase Bga35A | 622 | GH35 |  |
| Bpr_I2006 | beta-galactosidase Bga35B | 738 | GH35 |  |
| Bpr_I0788 | alpha-galactosidase Aga36A | 827 | GH36 |  |
| Bpr_I2883 | alpha-galactosidase Aga36B | 618 | GH36 |  |
| Bpr_III065 | alpha-galactosidase Aga36C | 730 | GH36 |  |
| Bpr_III017 | alpha-mannosidase man38A | 1067 | GH38 |  |
| Bpr_III060 | beta-xylosidase Xyl39A | 500 | GH39 |  |
| Bpr_I0036 | xylosidase/arabinofuranosidase Xsa43G | 316 | GH43 |  |
| Bpr_I0301 | xylosidase/arabinofuranosidase and esterase Xsa43H | 970 | GH43, CE10 |  |
| Bpr_I1584 | xylosidase/arabinofuranosidase Xsa43B | 643 | GH43 | Ank (x2) |
| Bpr_I1585 | xylosidase/arabinofuranosidase Xsa43C | 571 | GH43 |  |
| Bpr_I1784 | xylosidase/arabinofuranosidase Xsa43I | 316 | GH43 |  |
| Bpr_I2257 | xylosidase/arabinofuranosidase Xsa43D | 575 | GH43 |  |
| Bpr_I2319 | xylosidase/arabinofuranosidase Xsa43E | 313 | GH43 |  |
| Bpr_I0017 | alpha-L-arabinofuranosidase Arf51B | 505 | GH51 |  |
| Bpr_I0329 | alpha-L-arabinofuranosidase Arf51A | 501 | GH51 |  |
| Bpr_I0177 | alpha-D-glucuronidase Agu67A | 668 | GH67 |  |
| Bpr_I0911 | alpha-L-rhamnosidase Rha78B | 513 | GH78 |  |
| Bpr_I1686 | alpha-L-rhamnosidase Rha78A | 753 | GH78 |  |
| Bpr_III158 | alpha-L-rhamnosidase Rha78D | 724 | GH78 |  |
| Bpr_I2145 | unsaturated glucuronyl hydrolase Ugl88A | 411 | GH88 |  |
| Bpr_I2515 | unsaturated glucuronyl hydrolase Ugl88B | 402 | GH88 |  |
| Bpr_I2447 | cellobiose phosphorylase Cbp94A | 817 | GH94 | CBM_X |
| Bpr_III152 | cellobiose phosphorylase Cbp94B | 902 | GH94 |  |
| Bpr_III163 | glycoside hydrolase family 95 Gh95A | 714 | GH95 |  |
| Bpr_I0233 | unsaturated rhamnogalacturonyl hydrolase Gh105A | 349 | GH105 |  |
| Bpr_I2500 | unsaturated rhamnogalacturonyl hydrolase Gh105B | 380 | GH105 |  |
| Bpr_III020 | unsaturated rhamnogalacturonyl hydrolase Gh105C | 363 | GH105 |  |
| Bpr_I0232 | lacto-N-biose phosphorylase | 720 | GH112 |  |
| Bpr_I0179 | alpha-glucuronidase Gh115A | 987 | GH115 |  |
| Bpr_I1081 | alpha-glucuronidase Gh115B | 1039 | GH115 |  |
| Bpr_I0687 | glycoside hydrolase | 780 |  |  |
| Bpr_I2715 | glycoside hydrolase | 648 |  |  |
| Bpr_I0113 | xylosidase | 672 |  |  |
| Bpr_I1754 | xylosidase | 906 |  |  |
| Bpr_I1368 | feruloyl esterase Est1B | 284 | CE1 |  |
| Bpr_I2662 | carbohydrate esterase family 1 Est1D | 234 | CE1 |  |
| Bpr_I2870 | feruloyl esterase Est1E | 248 | CE1 |  |
| Bpr_I2939 | acetyl-xylan esterase Est2A | 376 | CE2 |  |
| Bpr_I0420 | polysaccharide deacetylase Est4A^4^ | 322 | CE4 |  |
| Bpr_I0439 | polysaccharide deacetylase Est4B^4^ | 631 | CE4, GT2 |  |
| Bpr_I1418 | polysaccharide deacetylase Est4D | 276 | CE4 |  |
| Bpr_I2536 | polysaccharide deacetylase Est4E4 | 249 | CE4 |  |
| Bpr_I1084 | pectin methylesterase Pme8A | 341 | CE8 |  |
| Bpr_I2926 | acetyl-xylan esterase Est10A | 276 | CE10 |  |
| Bpr_I1146 | carbohydrate esterase family 12 Est12A | 576 | CE12 |  |
| Bpr_I1586 | carbohydrate esterase family 12 Est12C | 240 | CE12 |  |
| Bpr_I0453 | N-acetylglucosaminyl-phosphatidylinositol de-N-acetylase family protein Est14A^4^ | 233 | CE14 |  |
| Bpr_I0174 | acetyl-xylan esterase | 669 |  |  |
| Bpr_I2118 | glycogen phosphorylase GlgP1 | 769 | GT35 |  |
| Bpr_I2847 | glycogen phosphorylase GlgP2 | 826 | GT35 |  |

1. TMH, transmembrane helices; CBM, carbohydrate binding module; DUF1083, domain of unknown function 1083 (PF06452); CW, putative cell wall binding repeat (PF01473); PUD, bacterial pullanase-associated domain (PF03714); Big4, bacterial Ig-like domain-group 4 (PF07532); FIVAR, uncharacterised sugar-binding domain (PF07554); fn3, fibronectin type III domain (PF00041); FG, FG-GAP repeat (PF01839); CelD, N-terminal Ig-like domain of cellulase (PF02927); Big2, bacterial Ig-like domain-group 2 (PF02368); Ank, ankyrin repeat (PF00023); CBM_X, putative carbohydrate binding domain (PF06204).
2. This CDS may also have glycoside hydrolase activity. It contains a glycoside hydrolase, family 2/20, immunoglobulin-like beta-sandwich domain (IPR013812) together with a FOG: Glucan-binding domain (YG repeat) (COG5236). This COG domain is also found in CDSs Bpr_I0190, Bpr_I2473 and Bpr_I2935.
3. These proteins do not contain signal peptide sequences but the presence of binding domains suggests that they function extracellularly. Bpr_I0544 contains a single transmembrane domain with most of the protein predicted to be outside the cell.
4. These CDSs are located within large clusters of EPS genes and are probably not involved in plant polysaccharide breakdown.
