# Supplementary material for: The Glycobiome of the Rumen Bacterium Butyrivibrio proteoclasticus B316T Highlights Adaptation to a Polysaccharide-Rich Environment
Source: PLoS One. 2010 Aug 3;5(8):e11942. doi: 10.1371/journal.pone.0011942 (PMC2914790; doi:10.1371/journal.pone.0011942)
Supplement: Table S2 — Diversity of plant polysaccharide degradation capabilities in rumen fibrolytic bacteria compared with the rumen metagenome. (0.03 MB DOC) [file pone.0011942.s005.doc]

Table S2. Diversity of plant polysaccharide degradation capabilities in rumen fibrolytic bacteria compared with the rumen metagenome.

| CAZy family | *B. proteoclasticus* B316 | *F. succinogenes* S85 | *R. flavefaciens* FD-1[16] | Rumen metagenome [8] |
| --- | --- | --- | --- | --- |
| Glycoside hydrolase | 34 | 25 | 25 | 38^1^ |
| Carbohydrate esterase | 7 | 6 | 7 | 5 |
| Polysaccharide lyase | 3 | 5 | 3 | 1 |
| Carbohydrate-binding module | 8 | 9 | 8 | 3 |
| Total | 52 | 45 | 43 | 47 |

1. The following glycoside hydrolase families were found in the rumen metagenome but not detected in B316, S85 or FD-1: GH4, GH15, GH33, GH92 and GH106.
