# Supplementary material for: The Glycobiome of the Rumen Bacterium Butyrivibrio proteoclasticus B316T Highlights Adaptation to a Polysaccharide-Rich Environment
Source: PLoS One. 2010 Aug 3;5(8):e11942. doi: 10.1371/journal.pone.0011942 (PMC2914790; doi:10.1371/journal.pone.0011942)
Supplement: Table S3 — Polysaccharide utilization loci (PUL) from the genome of B. proteoclasticus B316. (0.08 MB DOC) [file pone.0011942.s006.doc]

Table S3. Polysaccharide utilization loci (PUL) from the genome of *B. proteoclasticus* B316.

| Locus | Locus tag | Gene name |
| --- | --- | --- |
| PUL1 | Bpr_I0036  Bpr_I0037  Bpr_I0038 | xylosidase/arabinofuranosidase Xsa43G  two component system histidine kinase  two component system response regulator |
| PUL2 | Bpr_I0113  Bpr_I0114  Bpr_I0115  Bpr_I0116  Bpr_I0117 | xylosidase  transcriptional regulator LacI family  sugar ABC transporter permease protein  sugar ABC transporter permease protein  sugar ABC transporter substrate-binding protein |
| PUL3 | Bpr_I0170  Bpr_I0171  Bpr_I0172  Bpr_I0173  Bpr_I0174  Bpr_I0175  Bpr_I0176  Bpr_I0177  Bpr_I0178  Bpr_I0179  Bpr_I0180  Bpr_I0181  Bpr_I0182  Bpr_I0183  Bpr_I0184  Bpr_I0185  Bpr_I0186  Bpr_I0187  Bpr_I0188  Bpr_I0189  Bpr_I0190  Bpr_I0191  Bpr_I0192 | HD-GYP domain-containing protein  two component system histidine kinase/response regulator hybrid protein  HD-GYP domain-containing protein  xylulokinase XylB  acetyl-xylan esterase  glycoside hydrolase family 2 Gh2C  transcriptional regulator AraC family  alpha-D-glucuronidase Agu67A  transcriptional regulator AraC family  alpha-glucuronidase Gh115A  sugar ABC transporter permease protein  sugar ABC transporter permease protein  sugar ABC transporter substrate-binding protein  transposase IS200/IS605 family  beta-xylosidase Xyl3A  L-fucose isomerase related protein  D-isomer specfic 2-hydroxyacid dehydrogenase  **feruloyl esterase Est1C**  hypothetical secreted protein  transposase IS200/IS605 family  **lysozyme Lyc25C**  UTP-glucose-1-phosphate uridylyltransferase GalU  UDP-galactose 4-epimerase GalE |
| PUL4 | Bpr_I0232  Bpr_I0233  Bpr_I0234  Bpr_I0235  Bpr_I0236  Bpr_I0237 | lacto-N-biose phosphorylase  unsaturated rhamnogalacturonyl hydrolase Gh105A  transcriptional regulator AraC family  sugar ABC transporter permease protein  sugar ABC transporter permease protein  sugar ABC transporter substrate-binding protein |
| PUL5 | Bpr_I0299  Bpr_I0300  Bpr_I0301  Bpr_I0302  Bpr_I0303  Bpr_I0304  Bpr_I0305  Bpr_I0306  Bpr_I0307  Bpr_I0308  Bpr_I0309  Bpr_I0310  Bpr_I0311  Bpr_I0312  Bpr_I0313 | **endo-1,4-beta-glucanase/xylanase Cel5A**^1^  acetyltransferase GNAT family  xylosidase/arabinofuranosidase and esterase Xsa43H  **xylosidase/arabinofuranosidase Xsa43A**  transcriptional regulator AraC family  **endo-1,4-beta-xylanase Xyn10A**  sugar ABC transporter substrate-binding protein  sugar ABC transporter permease protein  sugar ABC transporter permease protein  NHL repeat-containing protein  conserved hypothetical transmembrane protein  conserved hypothetical secreted protein  sugar ABC transporter permease protein  sugar ABC transporter permease protein  sugar ABC transporter substrate-binding protein |
| PUL6 | Bpr_I0675  Bpr_I0676  Bpr_I0677  Bpr_I0678  Bpr_I0679  Bpr_I0680 | **xylosidase/arabinofuranosidase Xsa43F**  transcriptional regulator ArsR family  sugar ABC transporter substrate-binding protein  sugar ABC transporter permease protein  sugar ABC transporter permease protein  glycoside hydrolase family 2 Gh2D |
| PUL7 | Bpr_I0685  Bpr_I0686  Bpr_I0687  Bpr_I0689  Bpr_I0690  Bpr_I0691  Bpr_I0692  Bpr_I0693  Bpr_I0694  Bpr_I0695  Bpr_I0696  Bpr_I0697  Bpr_I0698  Bpr_I0699  Bpr_I0700  Bpr_I0701 | D-mannonate dehydratase UxuA  D-mannonate oxidoreductase UxuB  glycoside hydrolase  two component system response regulator (LytTR)  two component system histidine kinase  glycoside hydrolase family 2 Gh2A  sugar transporter GPH family  **beta-glucosidase Bgl3A**  hypothetical transmembrane protein  hypothetical secreted protein  transcriptional regulator LacI family  sugar ABC transporter substrate-binding protein  sugar ABC transporter permease protein  sugar ABC transporter permease protein  alpha-amylase Amy13F  glycoside hydrolase family 31 Gh31B |
| PUL8 | Bpr_I0906  Bpr_I0907  Bpr_I0908  Bpr_I0909  Bpr_I0910  Bpr_I0911  Bpr_I0912  Bpr_I0913  Bpr_I0914 | transcriptional regulator AraC family  sugar ABC transporter ATP-binding protein  sugar ABC transporter permease protein  sugar ABC transporter substrate-binding protein  sugar ABC transporter permease protein  alpha-L-rhamnosidase Rha78B  beta-N-acetylhexosaminidase Bhx3A  beta-phosphoglucomutase PgmB  GGDEF domain-containing protein |
| PUL9 | Bpr_I0929  Bpr_I0930  Bpr_I0931  Bpr_I0932  Bpr_I0933  Bpr_I0934  Bpr_I0935  Bpr_I0936  Bpr_I0937  Bpr_I0938 | 5-keto 4-deoxyuronate isomerase KduI  2-deoxy-D-gluconate 3-dehydrogenase KduD  2-keto-3-deoxygluconate 6-phosphate aldolase/2-keto-4-hydroxyglutarate aldolase  2-dehydro-3-deoxygluconokinase KdgK  HD-GYP domain-containing protein  transcriptional regulator LacI family  sugar ABC transporter permease protein  sugar ABC transporter permease protein  sugar ABC transporter substrate-binding protein  beta-galactosidase Bga35A |
| PUL10 | Bpr_I1081  Bpr_I1082  Bpr_I1083  Bpr_I1084 | alpha-glucuronidase Gh115B  hypothetical secreted protein  endo-1,4-beta-xylanase and esterase Xyn10D  pectin methylesterase Pme8A |
| PUL11 | Bpr_I1169  Bpr_I1170  Bpr_I1171  Bpr_I1172  Bpr_I1173  Bpr_I1174  Bpr_I1175  Bpr_I1176 | sugar ABC transporter substrate-binding protein  two component system histidine kinase  two component system response regulator (AraC)  xylose ABC transporter substrate binding protein  xylose ABC transporter substrate binding protein  xylose ABC transporter ATP-binding protein  xylose ABC transporter permease protein  EAL domain-containing protein |
| PUL12 | Bpr_I1232  Bpr_I1233  Bpr_I1234  Bpr_I1235  Bpr_I1236  Bpr_I1237 | glycogen debranching enzyme GlgX1  conserved hypothetical protein  two component system response regulator  two component system histidine kinase  hypothetical secreted protein  chemotaxis protein CheW |
| PUL13 | Bpr_I1317  Bpr_I1318  Bpr_I1319  Bpr_I1320  Bpr_I1321  Bpr_I1322 | sugar ABC transporter substrate-binding protein  sugar ABC transporter permease protein  sugar ABC transporter permease protein  conserved hypothetical transmembrane protein  two component system histidine kinase  two component system response regulator (AraC) |
| PUL14 | Bpr_I1583  Bpr_I1584  Bpr_I1585  Bpr_I1586  Bpr_I1587  Bpr_I1588  Bpr_I1589  Bpr_I1590  Bpr_I1591  Bpr_I1592  Bpr_I1593  Bpr_I1594  Bpr_I1595 | xylose isomerase domain-containing protein  xylosidase/arabinofuranosidase Xsa43B  xylosidase/arabinofuranosidase Xsa43C  carbohydrate esterase family 12 Est12C  sugar ABC transporter permease protein  sugar ABC transporter permease protein  sugar ABC transporter substrate-binding protein  altronate hydrolase UxaA  glucuronate isomerase UxaC  transcriptional regulator LacI family  cellodextrinase Cel9B  conserved hypothetical protein  carbohydrate kinase ROK family |
| PUL15 | Bpr_I1680  Bpr_I1681  Bpr_I1682  Bpr_I1683  Bpr_I1684  Bpr_I1685  Bpr_I1686  Bpr_I1687  Bpr_I1688  Bpr_I1689  Bpr_I1690 | GGDEF domain-containing protein  sugar fermentation stimulation protein SfsA  phosphotyrosine protein phosphatase  methyl-accepting chemotaxis protein McpI  xylose isomerase domain-containing protein  beta-glucosidase Bgl1A  alpha-L-rhamnosidase Rha78A  glycoside hydrolase family 2 Gh2E  sugar transporter GPH family  transcriptional regulator AraC family  transcriptional regulator AraC family |
| PUL16 | Bpr_I1699  Bpr_I1700  Bpr_I1701  Bpr_I1702  Bpr_I1703  Bpr_I1704  Bpr_I1705  Bpr_I1706  Bpr_I1707  Bpr_I1708  Bpr_I1709  Bpr_I1710 | glycoside hydrolase family 2 Gh2F  TPR domain-containing protein  transcriptional regulator AraC family  carbohydrate kinase PfkB family  sucrose-6-phosphate hydrolase Scr32A  glycoside hydrolase family 32 Gh32A  sugar ABC transporter substrate-binding protein  sugar ABC transporter permease protein  sugar ABC transporter permease protein  transcriptional regulator LacI family  sortase B family protein  **endo-1,4-beta-glucanase Cel5C** |
| PUL17 | Bpr_I1716  Bpr_I1717  Bpr_I1718  Bpr_I1719  Bpr_I1720 | two component system response regulator (AraC)  two component system histidine kinase  sugar ABC transporter permease protein  sugar ABC transporter permease protein  sugar ABC transporter substrate-binding protein |
| PUL18 | Bpr_I1754  Bpr_I1755  Bpr_I1756  Bpr_I1757 | xylosidase  hypothetical protein  GGDEF domain-containing protein  GGDEF/EAL/PAS domain-containing protein |
| PUL19 | Bpr_I1778  Bpr_I1779  Bpr_I1780  Bpr_I1781  Bpr_I1782  Bpr_I1783  Bpr_I1784 | rhamnulokinase RhaB  rhamnulose-1-phosphate aldolase RhaD  L-rhamnose isomerase RhaA  CAAX amino terminal protease family protein  aldose 1-epimerase family protein  transcriptional regulator AraC family  xylosidase/arabinofuranosidase Xsa43I |
| PUL20 | Bpr_I1838  Bpr_I1839  Bpr_I1840  Bpr_I1841  Bpr_I1842  Bpr_I1843  Bpr_I1844  Bpr_I1845  Bpr_I1846 | EAL domain-containing protein  conserved hypothetical protein  alpha-amylase Amy13C  PAP2 family protein  GDSL-family lipase/acylhydrolase  conserved hypothetical protein  **feruloyl esterase Est1A**  two component system histidine kinase  two component system response regulator |
| PUL21 | Bpr_I2003  Bpr_I2004  Bpr_I2005  Bpr_I2006  Bpr_I2007  Bpr_I2008  Bpr_I2009  Bpr_I2010 | EAL domain-containing protein  EAL domain-containing protein  conserved hypothetical protein  beta-galactosidase Bga35B  transcriptional regulator LacI family  sugar ABC transporter permease protein  sugar ABC transporter permease protein  sugar ABC transporter substrate-binding protein |
| PUL22 | Bpr_2095  Bpr_2096  Bpr_2097  Bpr_2098  Bpr_2099  Bpr_I2100  Bpr_I2101  Bpr_I2102  Bpr_I2103  Bpr_I2104  Bpr_I2105  Bpr_I2106 | beta-glucosidase Bgl3E  beta-glucosidase Bgl3D  GGDEF domain-containing protein  two component system histidine kinase  two component system response regulator (LytTR)  conserved hypothetical protein  chemotaxis protein CheC family  transcriptional regulator DeoR family  1-phosphofructokinase PfkB  PTS system IIABC fructose-specific family  PTS system HPr phosphocarrier  PTS system I PEP-phosphotransferase |
| PUL23 | Bpr_I2114  Bpr_I2115  Bpr_I2116  Bpr_I2117  Bpr_I2118  Bpr_I2119  Bpr_I2120 | HD-GYP/GAF domain-containing protein  metallo-beta-lactamase family protein  sugar ABC transporter substrate-binding protein  methyl-accepting chemotaxis protein McpL  glycogen phosphorylase GlgP1  transcriptional regulator LacI family  GGDEF/cache domain-containing protein |
| PUL24 | Bpr_2261  Bpr_2262  Bpr_2263  Bpr_2264  Bpr_2265  Bpr_2266 | sucrose phosphorylase Suc13P  conserved hypothetical protein  transcriptional regulator LacI family  sugar ABC transporter substrate-binding protein  sugar ABC transporter permease protein  sugar ABC transporter permease protein |
| PUL25 | Bpr_I2339  Bpr_I2340  Bpr_I2341  Bpr_I2342  Bpr_I2343  Bpr_I2344 | two component system response regulator (AraC)  two component system histidine kinase  sugar ABC transporter substrate-binding protein  sugar ABC transporter permease protein  sugar ABC transporter permease protein  sugar ABC transporter substrate-binding protein |
| PUL26 | Bpr_I2443  Bpr_I2444  Bpr_I2445  Bpr_I2446  Bpr_I2447 | sugar ABC transporter substrate-binding protein  sugar ABC transporter permease protein  sugar ABC transporter permease protein  transcriptional regulator LacI family  cellobiose phosphorylase Cbp94A |
| PUL27 | Bpr_I2681  Bpr_I2682  Bpr_I2683  Bpr_I2684  Bpr_I2685  Bpr_I2686  Bpr_I2687  Bpr_I2688 | sugar ABC transporter permease protein  sugar ABC transporter permease protein  sugar ABC transporter substrate-binding protein  alpha-amylase Amy13D  hypothetical secreted protein  transcriptional regulator LacI family  transcriptional regulator TetR family  transcriptional regulator AbrB family |
| PUL28 | Bpr_I2712  Bpr_I2713  Bpr_I2714  Bpr_I2715  Bpr_I2716 | sugar ABC transporter permease protein  sugar ABC transporter permease protein  sugar ABC transporter substrate-binding protein  glycoside hydrolase  transcriptional regulator LacI family |
| PUL29 | Bpr_I2867  Bpr_I2868  Bpr_I2869  Bpr_I2870 | two component system histidine kinase  two component system response regulator  ABC transporter ATP-binding protein  feruloyl esterase Est1E |
| PUL30 | Bpr_I2880  Bpr_I2881  Bpr_I2882  Bpr_I2883  Bpr_I2884 | transcriptional regulator ArsR family  HTH domain-containing protein  HTH/TPR domain-containing protein  alpha-galactosidase Aga36B  GGDEF domain-containing protein |
| PUL31 | Bpr_I2935  Bpr_I2936  Bpr_I2937  Bpr_I2938  Bpr_I2939 | **xylosidase/arabinofuranosidase Xsa43J**  transcriptional regulator LacI family  **glycoside hydrolase family 30 Gh30A**  oxidoreductase aldo/keto reductase family  acetyl-xylan esterase Est2A |
| PUL32 | Bpr_III015  Bpr_III016  Bpr_III017  Bpr_III018  Bpr_III019  Bpr_III020  Bpr_III021 | alpha-L-fucosidase Fuc29A  conserved hypothetical protein  alpha-mannosidase man38A  PHP domain-containing protein  transcriptional regulator AraC family  unsaturated rhamnogalacturonyl hydrolase Gh105C  carbohydrate kinase ROK family |
| PUL33 | Bpr_III199  Bpr_III200  Bpr_III201  Bpr_III202  Bpr_III203  Bpr_III204 | glycoside hydrolase family 31 Gh31E  sugar ABC transporter permease protein  sugar ABC transporter permease protein  sugar ABC transporter substrate-binding protein  two component system response regulator (AraC)  two component system histidine kinase |
| PUL34 | Bpr_III208  Bpr_III209 Bpr_III210 Bpr_III211 Bpr_III212 | **beta-N-acetylhexosaminidase Bhx3C**  beta-galactosidase Bga2B  hypothetical protein  GGDEF domain-containing protein  sugar ABC transporter substrate-binding protein |
| PUL35 | Bpr_III218  Bpr_III219  Bpr_III220 | **glycoside hydrolase family 3 Gh3A**  two component system histidine kinase  two component system response regulator (LytTR) |
| PUL36 | Bpr_III237  Bpr_III238  Bpr_III239  Bpr_III240  Bpr_III241  Bpr_III242  Bpr_III243  Bpr_III244  Bpr_III245  Bpr_III246  Bpr_III247  Bpr_III248  Bpr_III249  Bpr_III250 | beta-mannosidase Man2A  HTH domain-containing protein  response regulator domain-containing protein  sugar ABC transporter substrate-binding protein  sugar ABC transporter permease protein  sugar ABC transporter permease protein  sugar ABC transporter ATP-binding protein  sugar ABC transporter substrate-binding protein  two component system histidine kinase  two component system response regulator (AraC)  sortase domain-containing protein  sugar ABC transporter substrate-binding protein  GGDEF domain-containing protein  mannose-6-phosphate isomerase ManA |

1. Secreted polysaccharide degrading enzymes are shown in bold.
