# Supplementary material for: The Glycobiome of the Rumen Bacterium Butyrivibrio proteoclasticus B316T Highlights Adaptation to a Polysaccharide-Rich Environment
Source: PLoS One. 2010 Aug 3;5(8):e11942. doi: 10.1371/journal.pone.0011942 (PMC2914790; doi:10.1371/journal.pone.0011942)
Supplement: Table S4 — Exopolysaccharide biosynthesis loci (EPSL) from the genome of B. proteoclasticus B316. (0.07 MB DOC) [file pone.0011942.s007.doc]

Table S4. Exopolysaccharide biosynthesis loci (EPSL) from the genome of *B. proteoclasticus* B316.

| Locus | Locus tag | Gene name |
| --- | --- | --- |
| EPSL1 | Bpr_I0141  Bpr_I0142  Bpr_I0143  Bpr_I0144  Bpr_I0145  Bpr_I0146  Bpr_I0147  **Bpr_I0148**^1^  **Bpr_I0149**  **Bpr_I0150**  **Bpr_I0151**  **Bpr_I0152**  **Bpr_I0153**  Bpr_I0154  Bpr_I0155 | HTH domain-containing protein  hypothetical protein  conserved hypothetical protein  hydrolase beta-phosphoglucomutase family  nucleotidyl transferase  conserved hypothetical protein  nucleotidyl transferase  hypothetical protein  glycosyl transferase GT4 family  mannose-6-phosphate isomerase/mannose-1-phosphate guanylyl transferase  GDP-mannose 4,6-dehydratase Gmd  conserved hypothetical protein  hypothetical protein  polysaccharide ABC transporter permease protein  polysaccharide ABC transporter ATP-binding/permease protein |
| EPSL2 | Bpr_I0344  Bpr_I0345  Bpr_I0346  Bpr_I0347  Bpr_I0348  Bpr_I0349  Bpr_I0350  Bpr_I0351  Bpr_I0352  Bpr_I0353  Bpr_I0354  Bpr_I0355  Bpr_I0356  Bpr_I0357  Bpr_I0358  Bpr_I0359  Bpr_I0360  Bpr_I0361  Bpr_I0362  Bpr_I0363  Bpr_I0364  Bpr_I0365 | glycosyl transferase GT4 family  aminotransferase DegT/DnrJ/EryC1/StrS family  UDP-N-acetylglucosamine 2-epimerase  exopolysaccharide biosynthesis polyprenyl glycosylphosphotransferase  NAD dependent epimerase/dehydratase  glycosyl transferase GT4 family  NAD dependent epimerase/dehydratase  conserved hypothetical protein  glycosyl transferase GT4 family  aminotransferase DegT/DnrJ/EryC1/StrS family  conserved hypothetical transmembrane protein  conserved hypothetical transmembrane protein  conserved hypothetical transmembrane protein  glycosyl transferase GT2 family  rubredoxin Rub  glycosyl transferase GT4 family  asparagine synthase [glutamine-hydrolyzing] AsnB2  WxcM-like protein  rubrerythrin Rbr2  flavin reductase-like domain-containing protein  conserved hypothetical transmembrane protein  glycosyl transferase GT4 family |
| EPSL3 | **Bpr_I0378**  **Bpr_I0379**  **Bpr_I0380**  **Bpr_I0381**  **Bpr_I0382**  **Bpr_I0383**  **Bpr_I0384**  **Bpr_I0385**  **Bpr_I0386**  **Bpr_I0387**  **Bpr_I0388**  **Bpr_I0389**  **Bpr_I0390**  **Bpr_I0391**  **Bpr_I0392**  **Bpr_I0393**  **Bpr_I0394**  **Bpr_I0395**  **Bpr_I0396**  **Bpr_I0397**  **Bpr_I0398**  **Bpr_I0399**  **Bpr_I0400**  **Bpr_I0401**  **Bpr_I0402**  **Bpr_I0403**  **Bpr_I0404**  **Bpr_I0405**  **Bpr_I0406**  **Bpr_I0407**  **Bpr_I0408**  **Bpr_I0409**  **Bpr_I0410**  **Bpr_I0411**  **Bpr_I0412**  **Bpr_I0413**  **Bpr_I0414**  **Bpr_I0415**  **Bpr_I0416**  **Bpr_I0417**  **Bpr_I0418**  **Bpr_I0419**  **Bpr_I0420**  **Bpr_I0421**  **Bpr_I0422**  **Bpr_I0423**  **Bpr_I0424**  **Bpr_I0425**  **Bpr_I0426**  **Bpr_I0427**  **Bpr_I0428**  **Bpr_I0429**  **Bpr_I0430**  **Bpr_I0431**  **Bpr_I0432**  **Bpr_I0433**  **Bpr_I0434**  **Bpr_I0435**  **Bpr_I0436**  **Bpr_I0437**  **Bpr_I0438**  **Bpr_I0439**  **Bpr_I0440**  **Bpr_I0441**  **Bpr_I0442**  **Bpr_I0443**  **Bpr_I0444**  **Bpr_I0445**  **Bpr_I0446**  **Bpr_I0447**  **Bpr_I0448**  **Bpr_I0449**  **Bpr_I0450**  **Bpr_I0451**  **Bpr_I0452**  Bpr_I0453  Bpr_I0454  Bpr_I0455  Bpr_I0456 | conserved hypothetical protein  conserved hypothetical protein  ABC transporter ATP-binding/permease protein  glycosyl transferase GT4 family  glycosyl transferase GT11 family  aminotransferase DegT/DnrJ/EryC1/StrS family  glycosyl transferase GT2 family  acetyltransferase  glycosyl transferase  glycosyl transferase  conserved hypothetical protein  glycosyl transferase GT4 family  glycosyl transferase GT2 family  hypothetical transmembrane protein  glycosyl transferase GT4 family  glycosyl transferase GT2 family  polysaccharide biosynthesis protein  conserved hypothetical protein  NAD-dependent epimerase/dehydratase  acetyltransferase  glycosyl transferase GT4 family  conserved hypothetical protein  hypothetical transmembrane protein  acyltransferase  glycosyl transferase GT2 family  glycosyl transferase GT11 family  conserved hypothetical protein  glycosyl transferase GT4 family  oxidoreductase GFO/IDH/MOCA family  asparagine synthase [glutamine-hydrolyzing] AsnB3  glycosyl transferase GT4 family  glycosyl transferase GT2 family  acyltransferase  ABC transporter ATP-binding/permease protein  conserved hypothetical protein  glycosyl transferase GT11 family  hypothetical protein  nucleotidyl transferase  SAM-dependent methyltransferase  conserved hypothetical protein  AMP-binding enzyme  phosphopantetheine-binding protein  polysaccharide deacetylase Est4A  oxidoreductase GFO/IDH/MOCA family  oxidoreductase GFO/IDH/MOCA family  radical SAM domain-containing protein  polysaccharide biosynthesis protein  aminotransferase DegT/DnrJ/EryC1/StrS family  acetyltransferase  acetyltransferase  N-acetylneuraminate synthase NeuB (legionaminic acid synthase)  UDP-N-acetylglucosamine 2-epimerase  oxidoreductase GFO/IDH/MOCA family  oxidoreductase GFO/IDH/MOCA family  CMP-N-acetylneuraminic acid synthetase NeuA (CMP-legionaminic acid synthase)  asparagine synthase [glutamine-hydrolyzing] AsnB4  hypothetical transmembrane protein  asparagine synthase [glutamine-hydrolyzing] AsnB5  conserved hypothetical protein  acyltransferase  glycosyl transferase GT2 family  polysaccharide deacetylase Est4B  hypothetical transmembrane protein  hypothetical transmembrane protein  glycosyl transferase GT4 family  ABC transporter ATP-binding/permease protein  hypothetical protein  glycosyl transferase GT8 family  conserved hypothetical transmembrane protein  glycosyl transferase GT8 family  conserved hypothetical transmembrane protein  conserved hypothetical transmembrane protein  hypothetical protein  conserved hypothetical transmembrane protein  conserved hypothetical secreted protein  N-acetylglucosaminyl-phosphatidylinositol de-N-acetylase family protein Est14A  glycosyl transferase GT2 family  glycosyl transferase GT4 family  glycosyl transferase GT2 family |
| EPSL4 | Bpr_I0490  Bpr_I0491  Bpr_I0492  Bpr_I0493  Bpr_I0494  Bpr_I0495  Bpr_I0496  Bpr_I0497  Bpr_I0498  Bpr_I0499  Bpr_I0500  Bpr_I0501  Bpr_I0502  Bpr_I0503  Bpr_I0504  Bpr_I0505  Bpr_I0507  Bpr_I0508  Bpr_I0509  Bpr_I0510  Bpr_I0511  Bpr_I0512  Bpr_I0513  Bpr_I0514  Bpr_I0515  Bpr_I0516  Bpr_I0517  Bpr_I0518  Bpr_I0519  Bpr_I0520  Bpr_I0521  Bpr_I0522  Bpr_I0523  Bpr_I0524  Bpr_I0525  Bpr_I0526  Bpr_I0527  Bpr_I0528  Bpr_I0529  Bpr_I0530  Bpr_I0531  Bpr_I0532  Bpr_I0533  Bpr_I0534  Bpr_I0535  Bpr_I0536  Bpr_I0537  Bpr_I0538  Bpr_I0539  Bpr_I0540  Bpr_I0541  Bpr_I0542  Bpr_I0543  Bpr_I0544  Bpr_I0545  Bpr_I0546  Bpr_I0547  Bpr_I0548  Bpr_I0549  Bpr_I0550  Bpr_I0551  Bpr_I0552  Bpr_I0553  Bpr_I0554  Bpr_I0555  Bpr_I0556  Bpr_I0557  Bpr_I0558  Bpr_I0559  Bpr_I0560 | conserved hypothetical protein (CgeB)  conserved hypothetical protein (CgeB)  conserved hypothetical protein (CgeB)  glycosyl transferase GT2 family  conserved hypothetical protein  glycosyl transferase GT2 family  conserved hypothetical protein  glycosyl transferase GT11 family  glycosyl transferase GT2 family  glycosyl transferase GT2 family  acetyltransferase  conserved hypothetical protein  conserved hypothetical protein  glycosyl transferase  conserved hypothetical protein  glycosyl transferase GT2 family  conserved hypothetical protein (CgeB)  LicD family protein  LicD family protein  ribulose-phosphate 3-epimerase family protein  LicD family protein  conserved hypothetical protein  radical SAM domain-containing protein  glycosyl transferase GT26 family  polysaccharide biosynthesis protein (repeat unit polymerase)  undecaprenyl-phosphate glucose phosphotransferase  NAD-dependent epimerase/dehydratase  FAD dependent oxidoreductase  glycosyl transferase GT2 family  glycosyl transferase GT2 family  aminotransferase DegT/DnrJ/EryC1/StrS family  conserved hypothetical transmembrane protein  conserved hypothetical transmembrane protein  radical SAM domain-containing protein  glycosyl transferase GT2 family  polysaccharide biosynthesis protein  GDSL-family lipase/acylhydrolase  flavin containing amine oxidoreductase  glycosyl transferase GT2 family  acyltransferase MBOAT family  conserved hypothetical secreted protein  uridine kinase Udk  conserved hypothetical transmembrane protein  conserved hypothetical transmembrane protein  LicD family protein  aminotransferase  ornithine cyclodeaminase  acyltransferase MBOAT family  conserved hypothetical protein  conserved hypothetical protein  hypothetical protein  conserved hypothetical transmembrane protein  conserved hypothetical transmembrane protein  lysozyme Lyc25E  dTDP-glucose 4,6-dehydratase RfbB  glucose-1-phosphate thymidylyltransferase RfbA  hypothetical secreted protein  dTDP-4-dehydrorhamnose 3,5-epimerase RfbC  polysaccharide ABC transporter permease protein  polysaccharide ABC transporter ATP-binding protein  SAM-dependent methyltransferase  glycosyl transferase GT2 family  glycosyl transferase GT2 family  phosphoglucomutase/phosphomannomutase family protein  glycosyl transferase GT2 family  glycosyl transferase GT2 family  exopolysaccharide biosynthesis polyprenyl glycosylphosphotransferase  cell envelope-related transcriptional attenuator  glycosyl transferase GT2 family  RelA/SpoT domain-containing protein |
| EPSL5 | Bpr_I0708  Bpr_I0709  Bpr_I0710  **Bpr_I0711**  **Bpr_I0712**  **Bpr_I0713**  **Bpr_I0714**  **Bpr_I0715**  **Bpr_I0716**  **Bpr_I0717**  Bpr_I0718  Bpr_I0719 | HTH domain-containing protein  hypothetical protein  bacterial sugar transferase  glycosyl transferase GT4 family  asparagine synthase [glutamine-hydrolyzing] AsnB6  glycosyl transferase GT2 family  polysaccharide biosynthesis protein (repeat unit polymerase)  polysaccharide biosynthesis protein (flippase)  glycosyl transferase GT4 family  glycosyl transferase  NAD-dependent epimerase/dehydratase (frameshift)  NAD-dependent epimerase/dehydratase |
| EPSL6 | Bpr_I0755  Bpr_I0756  Bpr_I0757  Bpr_I0758  Bpr_I0759  Bpr_I0760  Bpr_I0761  Bpr_I0762 | HD-GYP/cache domain-containing protein  hypothetical secreted protein  hypothetical transmembrane protein  hypothetical protein  glycosyl transferase GT2 family  glycosyl transferase GT4 family  glycosyl transferase GT2 family  glycosyl transferase GT2 family |
| EPSL7 | Bpr_I0805  Bpr_I0806  Bpr_I0807  Bpr_I0808  Bpr_I0809  Bpr_I0810  Bpr_I0811  Bpr_I0812  **Bpr_I0813**  **Bpr_I0814**  **Bpr_I0815**  **Bpr_I0816**  **Bpr_I0817**  **Bpr_I0818**  **Bpr_I0819**  **Bpr_I0820** | polysaccharide export protein  sortase B family protein  cell envelope-related transcriptional attenuator  hypothetical protein  polysaccharide biosynthesis protein  polysaccharide biosynthesis protein  glycosyl transferase GT4 family  acetyltransferase  glycosyl transferase GT2 family  glycosyl transferase GT4 family  conserved hypothetical protein  coenzyme F420-reducing hydrogenase beta subunit  acetyltransferase  glycosyl transferase GT4 family  polysaccharide biosynthesis protein (repeat unit polymerase)  polysaccharide biosynthesis protein (flippase) |
| EPSL8 | Bpr_I1106  Bpr_I1107  **Bpr_I1108**  **Bpr_I1109**  **Bpr_I1110**  **Bpr_I1111**  **Bpr_I1112**  **Bpr_I1113**  **Bpr_I1114**  **Bpr_I1115**  **Bpr_I1116**  **Bpr_I1117**  **Bpr_I1118**  **Bpr_I1119**  **Bpr_I1120**  Bpr_I1121  Bpr_I1122  Bpr_I1123  Bpr_I1124 | resolvase family protein  HTH domain-containing protein  hypothetical protein  glycosyl transferase GT2 family  glycosyl transferase GT2 family  dTDP-4-dehydrorhamnose 3,5-epimerase RfbC  glycosyl transferase GT4 family  radical SAM domain-containing protein  NAD-dependent epimerase/dehydratase  SAM-dependent methyltransferase  NAD-dependent epimerase/dehydratase  glucose-1-phosphate cytidylyltransferase RfbF1  glucose-1-phosphate cytidylyltransferase RfbF2  aminotransferase DegT/DnrJ/EryC1/StrS family  NUDIX domain-containing protein  conserved hypothetical protein  polysaccharide ABC transporter permease protein  polysaccharide ABC transporter ATP-binding/permease protein  conserved hypothetical protein |
| EPSL9 | Bpr_I2179  Bpr_I2180  **Bpr_I2181**  **Bpr_I2182**  **Bpr_I2183**  **Bpr_I2184**  **Bpr_I2185**  **Bpr_I2186**  **Bpr_I2187**  **Bpr_I2188**  **Bpr_I2189** | glycosyl transferase GT2 family (truncated pseudogene)  radical SAM domain-containing protein  glycosyl transferase  conserved hypothetical protein  glycosyl transferase GT2 family  glycosyl transferase GT2 family  glycosyl transferase GT2 family  radical SAM domain-containing protein  conserved hypothetical protein  hypothetical protein  acetyltransferase |
| EPSL10 | Bpr_I2305  Bpr_I2306  Bpr_I2307  Bpr_I2308  Bpr_I2309  Bpr_I2310  Bpr_I2311  Bpr_I2312  Bpr_I2313  Bpr_I2314  Bpr_I2315 | UDP-galactopyranose mutase Glf  transcriptional regulator  hypothetical protein  glycosyl transferase GT2 family  hypothetical protein  NAD-dependent epimerase/dehydratase  aminotransferase DegT/DnrJ/EryC1/StrS family  CDP-glucose 4,6-dehydratase RfbG  glycosyl transferase GT2 family  hypothetical secreted protein  hypothetical transmembrane protein |
| EPSL11 | Bpr_I2380  Bpr_I2381  Bpr_I2382  Bpr_I2383  Bpr_I2384  Bpr_I2385  Bpr_I2386  Bpr_I2387  Bpr_I2388  Bpr_I2389  Bpr_I2390  Bpr_I2391  Bpr_I2392  Bpr_I2393  Bpr_I2394  Bpr_I2395  Bpr_I2396  Bpr_I2397  Bpr_I2398 | asparagine synthase [glutamine-hydrolyzing] AsnB8  conserved hypothetical protein  conserved hypothetical protein  glycosyl transferase GT2 family  conserved hypothetical protein  undecaprenyl-phosphate glucose phosphotransferase  glycosyl transferase GT28 family  polysaccharide biosynthesis protein  diaminopimelate epimerase DapF  SAM-dependent methyltransferase  transporter small multi-drug export family  histidinol-phosphate aminotransferase HisC  hypothetical secreted protein  sortase B family protein  cell envelope-related transcriptional attenuator  hypothetical secreted protein  polysaccharide biosynthesis protein  polysaccharide export protein  polysaccharide biosynthesis protein |
| EPSL12 | **Bpr_I2412**  **Bpr_I2413**  **Bpr_I2414**  **Bpr_I2415**  **Bpr_I2416**  **Bpr_I2417**  **Bpr_I2418**  **Bpr_I2419**  **Bpr_I2420**  **Bpr_I2421**  **Bpr_I2422**  **Bpr_I2423**  **Bpr_I2424**  **Bpr_I2425** | polysaccharide biosynthesis protein (flippase)  nucleotidyl transferase  GHMP kinase family protein  coenzyme F420-reducing hydrogenase beta subunit  polysaccharide pyruvyl transferase  glycosyl transferase GT2 family  CotH family protein  VanZ-like family protein  hypothetical transmembrane protein  glycosyl transferase GT2 family  polysaccharide export protein  acyltransferase  glycosyl transferase  acyltransferase |
| EPSL13 | Bpr_I2516  Bpr_I2517  Bpr_I2518  Bpr_I2519  Bpr_I2520  Bpr_I2521  Bpr_I2522  Bpr_I2523  Bpr_I2524  Bpr_I2525  Bpr_I2526  Bpr_I2527  Bpr_I2528  Bpr_I2529  Bpr_I2530  Bpr_I2531  Bpr_I2532  Bpr_I2533  Bpr_I2534  Bpr_I2535  Bpr_I2536  Bpr_I2537  Bpr_I2538  Bpr_I2539  Bpr_I2540  Bpr_I2541  Bpr_I2542  Bpr_I2543  Bpr_I2544  Bpr_I2545  Bpr_I2546  Bpr_I2547  Bpr_I2548  Bpr_I2549  Bpr_I2550  Bpr_I2551  Bpr_I2552  Bpr_I2553  Bpr_I2554  Bpr_I2555  Bpr_I2556  Bpr_I2557  Bpr_I2558  Bpr_I2559  Bpr_I2560  Bpr_I2561  Bpr_I2562  Bpr_I2563  Bpr_I2564  Bpr_I2565  Bpr_I2566  Bpr_I2567  Bpr_I2568  Bpr_I2569  Bpr_I2570  Bpr_I2571  Bpr_I2572  Bpr_I2573  Bpr_I2574  Bpr_I2575  Bpr_I2576  Bpr_I2577  Bpr_I2578  Bpr_I2579  Bpr_I2580  Bpr_I2581  Bpr_I2582 | polysaccharide biosynthesis protein (flippase)  glycosyl transferase GT11 family  polysaccharide pyruvyl transferase  glycosyl transferase GT2 family  conserved hypothetical transmembrane protein  polysaccharide biosynthesis protein (repeat unit polymerase)  glycosyl transferase GT17 family  glycosyl transferase GT2 family  conserved hypothetical protein  glycosyl transferase GT11 family  ABC transporter ATP-binding/permease protein  glycosyl transferase GT11 family  asparagine synthase [glutamine-hydrolyzing] AsnB9  conserved hypothetical secreted protein  acyltransferase MBOAT family  glycosyl transferase GT4 family  asparagine synthase [glutamine-hydrolyzing] AsnB10  glycosyl transferase GT4 family  glycosyl transferase GT4 family  glycosyl transferase GT4 family  polysaccharide deacetylase Est4E  NAD-dependent epimerase/dehydratase  nucleotide sugar dehydrogenase  glycosyl transferase GT4 family  conserved hypothetical protein  pyridoxal phosphate-dependent deaminase  bacterial sugar transferase  aminotransferase DegT/DnrJ/EryC1/StrS family  polysaccharide biosynthesis protein  hypothetical transmembrane protein  conserved hypothetical transmembrane protein  glycosyl transferase GT2 family  acetyltransferase GNAT family  conserved hypothetical transmembrane protein  TDP-4-keto-6-deoxy-D-glucose transaminase  GtrA-like protein  hypothetical transmembrane protein  conserved hypothetical transmembrane protein  ABC transporter ATP-binding/permease protein  glycosyl transferase GT28 family  CMP-N-acetylneuraminic acid synthetase NeuA  glycosyl transferase GT2 family  glycosyl transferase GT28 family  N-acetylneuraminate synthase NeuB  acetyltransferase GNAT family  lipooligosaccharide sialyltransferase  polysaccharide biosynthesis protein  conserved hypothetical transmembrane protein  conserved hypothetical transmembrane protein  glycosyl transferase GT2 family  conserved hypothetical transmembrane protein  glycosyl transferase GT4 family  glycosyl transferase GT4 family  glycosyl transferase GT2 family  polysaccharide biosynthesis protein (repeat unit polymerase)  glycosyl transferase GT2 family  polysaccharide export protein  exopolysaccharide biosynthesis polyprenyl glycosylphosphotransferase  glycosyl transferase GT2 family  glycosyl transferase GT2 family  transcriptional regulator AraC family  GDP-mannose 4,6-dehydratase Gmd  NAD-dependent epimerase/dehydratase  SCP-like extracellular protein  conserved hypothetical transmembrane protein  conserved hypothetical transmembrane protein  dTDP-4-dehydrorhamnose reductase RfbD |

1. CDSs with a lower G+C% than the genome average are shown in bold.
